# The structure, organization and radiation of *Sadhu *non-long terminal repeat retroelements in *Arabidopsis *species

**DOI:** 10.1186/1759-8753-1-10

**Published:** 2010-03-01

**Authors:** Sanjida H Rangwala, Eric J Richards

**Affiliations:** 1 Department of Biology, Washington University in St Louis, St Louis, MO, USA; 2 Department of Genetics, University of Pennsylvania School of Medicine, Philadelphia, PA, USA; 3 Boyce Thompson Institute for Plant Research, Cornell University, Ithaca, NY, USA

## Abstract

**Background:**

*Sadhu *elements are non-autonomous retroposons first recognized in *Arabidopsis thaliana*. There is a wide degree of divergence among different elements, suggesting that these sequences are ancient in origin. Here we report the results of several lines of investigation into the genomic organization and evolutionary history of this element family.

**Results:**

We present a classification scheme for *Sadhu *elements in *A. thaliana*, describing derivative elements related to the full-length elements we reported previously. We characterized *Sadhu5 *elements in a set of *A. thaliana *strains in order to trace the history of radiation in this subfamily. Sequences surrounding the target sites of different *Sadhu *insertions are consistent with mobilization by LINE retroelements. Finally, we identified *Sadhu *elements grouping into distinct subfamilies in two related species, *Arabidopsis arenosa *and *Arabidopsis lyrata*.

**Conclusions:**

Our analyses suggest that the *Sadhu *retroelement family has undergone target primed reverse transcription-driven retrotransposition during the divergence of different *A. thaliana *strains. In addition, *Sadhu *elements can be found at moderate copy number in three distinct *Arabidopsis *species, indicating that the evolutionary history of these sequences can be traced back at least several millions of years.

## Background

We previously reported a novel family of *Arabidopsis *retroposons, *Sadhu *[1]. The typical *Sadhu *element contains a poly(A) tract and is flanked by a direct 7 to 16 base pair (bp) target site duplication (TSD). Similar to small interspersed nuclear elements (SINEs), *Sadhu *elements are non-protein coding and do not contain long terminal repeats (LTRs); they are therefore expected to be non-autonomous. Although plant SINEs are thought to be mobilized by autonomous long interspersed nuclear elements (LINEs), the source of the transposase for *Sadhu *is not clear.

Structurally, *Sadhu *elements resemble SINEs (non-coding, poly(A) tract), but unlike known SINEs, they do not contain sequence similarity to known non-coding RNAs (for example, 5SrRNA, tRNA) [2]. Nor do *Sadhu *elements carry conserved sequences similar to RNA polymerase II TATA boxes or RNA polymerase III promoter motifs (for example, A and B boxes). However, *Sadhu *elements share a motif near the 5' end (consensus 5' CAATCGTTSC 3') and an approximately 20 bp polypyrimidine region that we hypothesize might attract GAGA-repeat binding transcription factors [3-5]. *Sadhu *elements in different *Arabidopsis thaliana *accessions are expressed, often at high levels. Sense transcription begins at or near the start of the element [6], consistent with the hypothesis that these elements carry their own internal promoter sequences. Expression can also occur in the antisense direction, presumably from promoters in the flanking DNA sequence. Whether sense or antisense, transcription of *Sadhu *elements is epigenetically regulated; silenced elements are associated with cytosine methylation and packaged in chromatin containing the dimethylated isoform of lysine 9 of histone H3 [1,6]. There is variation in the modes of silencing of various *Sadhu *family members highlighted by differential susceptibility to epigenetic modifier mutations and distinct cytosine methylation profiles. These findings suggest that *Sadhu *elements are silenced independently and individually, not coordinately [6]. For these diverse reasons, *Sadhu *represents a unique family of non-LTR retroelements.

Related families of the same transposable element class can often be detected by sequence similarity in widely divergent species (see for example, [7,8]). *Sadhu *elements within *A. thaliana *are highly divergent in terms of nucleotide sequence, with an average pairwise identity of less than 75%, suggestive of an ancient origin. However, these sequences cannot be identified in any of the current public genome databases outside of the Brassicaceae. There are only 39 *Sadhu*-related sequences in the *A. thaliana *genome, showing a dispersed distribution pattern across all five chromosomes. This moderate copy number is typical of *Arabidopsis *non-LTR retroelements: there are approximately 130 SINE elements in the *A. thaliana *reference genome and less than 1,500 LINEs [9]. The relatively low copy number of non-LTR retroelements in *A. thaliana *suggests that the transposition rate of these elements is low and/or that new insertions have been effectively removed during the evolutionary history of the species.

Here, we describe a classification scheme for this retroelement family. In addition, we investigate the organization and radiation of *Sadhu *sequences both in different *A. thaliana *accessions and related *Arabidopsis *species.

## Results and Discussion

### Classification of *Sadhu *elements

**Figure 1:**
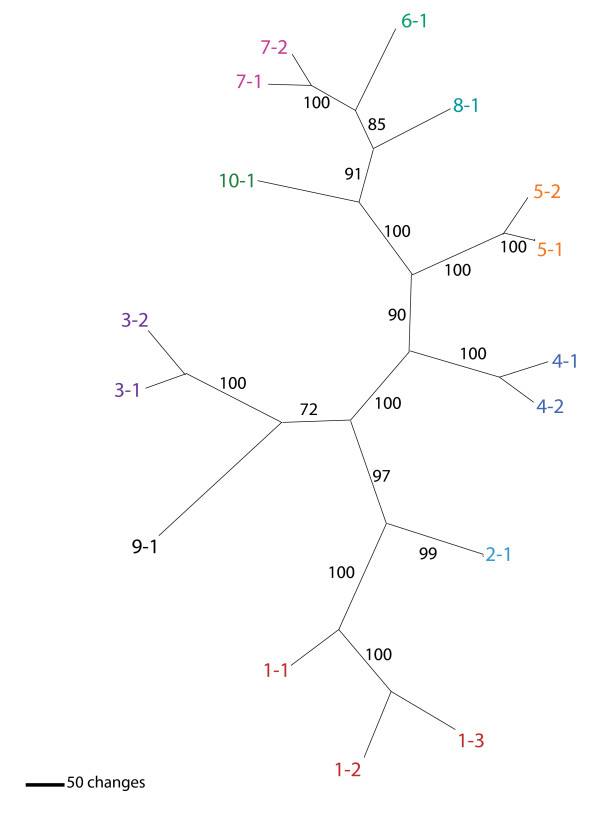
**Phylogenetic analysis of *Arabidopsis thaliana Sadhu *sequences**. Maximum parsimony phylogram of full-length *Sadhu *elements. Taxa are color coded according to ontological grouping. See Table 1 for gene ID numbers corresponding to *Sadhu *numbers. Bootstrap values (percentages) were calculated from 500 bootstrap replicates.

We designed a classification scheme for *Sadhu *elements reflecting the phylogenetic grouping of these elements into 10 distinct subfamilies in the *A. thaliana *genome (Table 1, Figure 1, Additional file 1) [1]. Table 1 lists the new nomenclature side by side with locus ID numbers (for full-length elements) or locus position (for partial elements). *Sadhu *elements that extend from the 5' conserved motif 5' CAATCGTTSC 3' to a 3' poly(A) tract approximately 900 bp downstream have been designated 'full length'. Full-length elements on the same branch of the phylogeny share a family name (*Sadhu#*), but have different element names (*SadhuX-#*). Elements that closely align (>75% identity) to a unique full-length element are designated 'd' indicating derived; for example, *Sadhu5-1d1 *is likely to be derived from *Sadhu5-1*. *Sadhu*-related sequences that are not similar to a unique full-length element are assigned to the nearest full-length element on a pairwise BLAST search with the designation 'L' for 'like' (for example, *Sadhu3L*). See Additional file 1 for divergence matrices among elements within different subfamilies and among subfamilies.

**Table 1:** *Sadhu*-related sequences in *Arabidopsis thaliana*.

*Sadhu *number	ID number (or position)	Nucleotide position in *A. thaliana *genome (TAIR 9.0)
1-1	At2g10410	Chr2: 4014110-4013202
1-2	At1g30835	Chr1: 10967854-10966931
1-3	At5g28626	Chr5: 10632245-10632826; 10633568-10633948^a^
1L1	At1g66795	Chr1: 24926769-24927016
2-1	At1g35112	Chr1: 12841125-12840206
2-1d1	At2g18535	Chr2: 8048795-8048610
3-1	At3g44042	Chr3: 15825096-15824141
3-2	At3g42658	Chr3: 14761424-14760388
3-1d1	At2g21905	Chr2: 9345876-9346247
3-1d2	At5g03205	Chr5: 762065-761915
3L1	At4g04925	Chr4: 2506188-2506806
4-1	At5g28913	Chr5: 10934749-10933814
4-2	At1g03420	Chr1: 846815-847698
4-2d1	At2g05027	Chr2: 1781076-1781386
5-1	At4g01525	Chr4: 660768-661723
5-1d1	At1g18195	Chr1: 6262595-6263382
5-1d2	At4g00953	Chr4: 410383-411018
5-2	At5g27927	Chr5: 9957820-9956864
6-1	At3g02515	Chr3: 525338-526263
6-1d1	At5g42095	Chr5: 16845951-16846349
6-1d2	At5g44565	Chr5: 17981087-17980529
6-1d3	At5g42237	Chr5: 16987448-16988447
6L1	At2g10935	Chr2: 4312891-4312205
7-1	At3g13438	Chr3: 4377991-4377083
7-2	At3g31442	Chr3: 12807354-12806392
7L1	At1g36745	Chr1: 13912120-13913031
7L2	At3g61625	Chr3: 22815058-22814684
7L3	At5g52140	Chr5: 21206508-21206698
8-1	At1g50735	Chr1: 18811080-18810175
8L1	At5g38915	Chr5: 15597647-15597844
8L2	At2g24745	Chr2: 10540693-10541337
8L3	At1g52615	Chr1: 19607182-19606826
9-1	At1g44935	Chr1: 16904928-16905344
9L1	At1g32455	Chr1: 11733481-11733785
9L2	At1g69365	Chr1: 26083199-26083479
10-1	At3g58865	Chr3: 21776975-21777729
10L1	At5g46395	Chr5: 18836667-18837050
10L2	At5g42945	Chr5: 17240081-17240294
10L3	At1g35255	Chr1: 12935906-12936263

^a^Position is discontinuous due to insertion of AtLANTYS2_LTR sequence.

Chr = chromosome.

### Partial *Sadhu *elements

**Figure 2:**
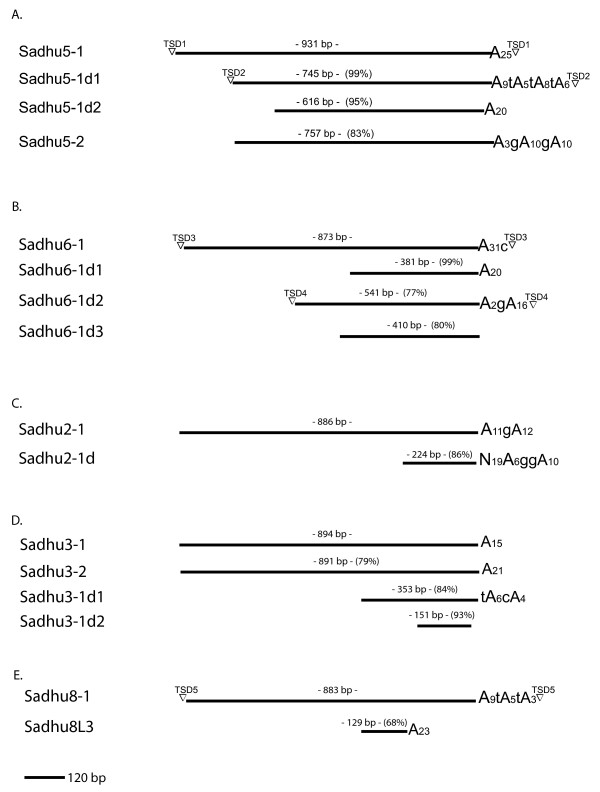
**Schematic alignment of selected *Sadhu *subfamilies in strain Col**. TSD sequences are different at different elements. Sizes of TSDs: TSD1, 11 base pairs (bp); TSD2, 12 bp; TSD3, 12 bp; TSD4, 10 bp; TSD5, 13 bp. Percentages correspond to sequence identity to the longest element in the subfamily. Sizes marked above each line represent positions relative to the gapped alignment and might be slightly different from the nucleotide length of element. (a) *Sadhu5*; (b) *Sadhu6*; (c) *Sadhu2*; (d) *Sadhu3*; (e) *Sadhu8-1 *versus *Sadhu8L3*. TSD = target site duplication.

The *Sadhu2*, *Sadhu3*, *Sadhu4*, *Sadhu5*, and *Sadhu6 *subfamilies feature derivative sequences that are greater than 80% identical to a particular full-length element (Figure 2, Table 1, Additional file 1). Many of the partial elements sequences are 5' truncated: that is, the region of similarity shared with the most closely related full-length element does not extend to the 5' end, but contains remnants of 3' poly(A) tracts (recognizably A-rich regions) and, in some cases, flanking direct repeats that represent TSDs. This pattern is consistent with abortive retrotransposition. Other partial sequences align to internal sections of full-length elements. In the case of *Sadhu2-1d*, a 3' poly(A) tract is detectable, but is preceded by a stretch of DNA sequence (19 bp) that does not align to the prospective progenitor *Sadhu *element (Figure 2c; *Sadhu7L1 *and *Sadhu10L3 *also have this structure). This type of chimeric retrotransposon structure can result from template switching during retrotransposition [10,11]. In contrast, the *Sadhu8L3 *derivative terminates in a poly(A) tract at a position earlier than its closest full-length element (Figure 2e). This structure might arise from abortive transcription and early polyadenylation of the precursor sequence or through subsequent internal deletion of the element. If partial elements arose by segmental duplication, we would expect to see DNA sequence similarity extending beyond the *Sadhu*-related sequence. However, none of the *Sadhu *elements in the Columbia (Col) reference genome shares significant sequence similarity in flanking genomic regions with their derivative elements. Therefore, it is more likely that the partial elements are remnants of ancestral retrotransposition followed by template switching, deletion and/or divergence.

### Radiation of the *Sadhu5 *subfamily in *A. thaliana*

A comparison of the genome sequences of two *Arabidopsis *strains, Col and Ler, revealed over 150 indels caused by differential activity of transposable elements between the strains [12]. We previously reported that several *Sadhu *elements from different subfamilies are also polymorphic in terms of presence/absence among different *Arabidopsis *strains [1,6]. Below, we examine closely related elements from a single subfamily in a set of 24 *A. thaliana *strains in order to trace the retrotranspositional history of these elements. The *Sadhu5 *subfamily contains four elements that are all greater than 80% identical to one another in the Col reference genome and close to full-length or full-length (>600 bp) (Figure 2a). *Sadhu5-1 *and *Sadhu5-2 *are 83% identical to one another, while the two derivative elements, *Sadhu5-1d1 *and *Sadhu5-1d2*, are greater than 95% identical to *Sadhu5-1*. This family therefore represents a closely related group of sequences that might have expanded during the recent evolutionary history of the species.

We began by examining the *Sadhu5-2 *element. A polymerase chain reaction (PCR) product corresponding to an internal region of this element was present in every strain examined (Table 2). We investigated whether *Sadhu5-2 *elements in different strains were present in the same genomic location: using an outward facing forward primer in the element and reverse primers designed based on the Col reference genome 5' and 3' adjacent sequence, we attempted to amplify PCR products spanning the flanks of the elements. In every case, we were successful in amplifying products of the expected size (Table 2). Therefore, it is likely that *Sadhu5-2 *represents a single insertion event in the ancestor of the *A. thaliana *lineage.

**Table 2:** Distribution of *Sadhu5 *subfamily members in natural strains.

Accession number	Stock number	*Sadhu5-1*			*Sadhu5-1d1*			*Sadhu5-1d2*			*Sadhu5-2*		
		
		Int	5'	3'	Int	5'	3'	Int	5'	3'	Int	5'	3'
Br-0	CS22628	ES			ES			X	X	X	X	X	X
Bur-0	CS22656				X	X	X	X	X	X	X	X	X
C24	CS22620				ES			X	X	X	X	X	X
Col	Lehle WT-2	X	X	X	X	X	X	X	X	X	X	X	X
Ct-1	CS22639	ES			ES			X	X	X	X	X	X
Cvi	Lehle WT-18	X	X*	X*	X	X*	X*	X	X	X	X	X	X
Cvi-0	CS22614				X	X	X	X	X	X	X	X	X
Fei-0	CS22645				ES			X	X	X	X	X	X
Hi-0	CS6736	X		X	ES			X	X	X	X	X	
Kn-0	CS6762	X	Short	X	ES			X	X	X	X	X	X
Kondara	CS22651	X	Long	X							X	X	X
Kz-1	CS22606	X	X*	X*				X*	X	X	X	X	X
Ler	Lehle WT-4	X			ES			X	X	X	X	X*	
N13	CS22491	X	X*	X	ES			X	X	X	X	X	X
Po-0	CS6839	X	X*	X*	ES			X	X	X	X	X	X
Pro-0	CS22649	X	X*	X				X*	X	X	X	X	X
Pu2-7	CS22592	X	X*	X	ES			X	X	X	X	X	X
Ra-0	CS22632	X	X	X				X	X	X	X	X	X
Tamm-27	CS22605	X	X	X	ES						X	X	X
Ts-1	CS22647				ES			X	X	X	X	X	X
Tsu-1	CS22641	X	X	X	ES			X	X	X	X	X	X
Van-0	CS22627	X	X	X				X	X	X	X	X	X
Wei-0	CS22622	X	X	X	ES			X*	X	X	X	X	X
Ws-2	CS22659	X	Long	X				X	X	X	X	X	X

Empty cells signify no PCR product amplified with the corresponding primers.

3' = PCR product with one primer located in the 3' flank and the other in the element; 5' = PCR product with one primer located in the 5' flank and the other in the element; ES = negative for int PCR, but empty site amplified with 5' and 3' flanking primers; int = internal PCR product, both primers located within the element; PCR = polymerase chain reaction; X* = PCR product with more distal but not with more proximal primers.

**Figure 3:**
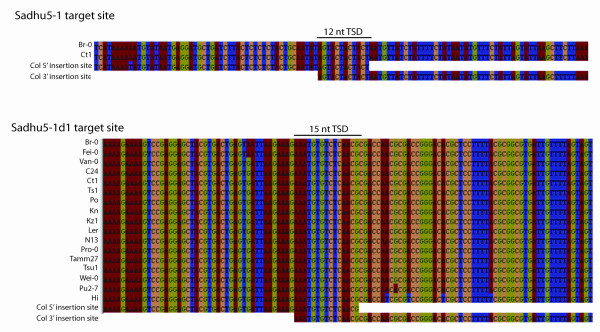
**Empty sites detected in *Arabidopsis thaliana *strains at positions occupied in Col by (a) *Sadhu5-1 *and (b)*Sadhu5-1d1***. Multiple sequence alignments of Col 5' and 3' sequences flanking the site of insertion along with sequences of strains that do not contain the insertion. Sequences corresponding to *Sadhu *element insertions have been removed. Genbank accession numbers for *Sadhu5-1 *sequences are EF535531 and EF535532. Genbank accession numbers for *Sadhu5-1d1 *sequences are EF535533, EF535534, EF535535, EF535536, EF535537, EF535538, EF535539, EF535540, EF535541, EF535542, EF535543, EF535544, EF535545, EF535546, EF535547, EF535548, and EF535549.

In contrast to our finding for *Sadhu5-2*, we were unable to amplify PCR products from several strains using primers specific to the *Sadhu5-1*, *Sadhu5-1d1 *or *Sadhu5-1d2 *insertion sites in the Col strain (Table 2). To investigate the structure of putative deletions or 'empty' sites for these elements, we amplified PCR products from these strains using primers located 5' and 3' of the element in the Col reference genome. We identified 2 strains for *Sadhu5-1 *and 17 strains for *Sadhu5-1d1 *that amplified a specific, shorter PCR product than would be predicted from the reference genome. We obtained DNA sequence for these PCR products: in every case, there was a clean retrotransposition 'empty site', with a single, identical copy of the target site duplication of the element in strain Col (Figure 3). The structure of the 'empty' versus the 'filled' sites are typical of retroelements that undergo target primed reverse transcription (TPRT) [13]. The Col strain carries the most common haplotype for the region surrounding the *Sadhu5-1d1 *insertion (Figure 3). Therefore, the most parsimonious explanation is that the element inserted relatively recently in the history of these strains, after the divergence of different haplotypes in this region.

The identification of clean presence/absence polymorphisms among *Arabidopsis *strains also lends support to the model that *Sadhu5-1 *and *Sadhu5-1d1 *are relatively recent retrotransposition events. In contrast, we could not find polymorphic insertion sites for *Sadhu5-1d2 *and *Sadhu5-2*, suggesting that these elements represent older, ancestral insertion events. *Sadhu5-2 *appears to be a truncated retrotransposition product relative to *Sadhu5-1*, as it is missing sequence that would align with the 5' portion of *Sadhu5-1 *(Figure 2a). Therefore, while the *Sadhu5-2 *sequence itself appears more prevalent than *Sadhu5-1*, the latter element could not be derived by retrotransposition or gene duplication from the former without invoking a subsequent deletion of the 5' region of the element, which is unlikely given that the same structure appears to exist in all strains based on PCR of the flanking regions (Table 2). An alternate hypothesis is that the full-length ancestor to this subfamily has been deleted or lost from the *A. thaliana *Col reference strain.

### Target site consensus

TSDs are typical of most transposable elements. Non-LTR retroelements mobilized by the LINE enzymatic machinery feature TSDs of 7 to 20 bp in length. These TSDs result from the target primed reverse transcription mechanism, where two staggered cuts are made on the target strand [13]. In mammals, the consensus for the LINE 5' endonuclease cleavage site contains two thymines, whereas the duplicated target site often starts with a string of four adenines [14-16]. This string of adenines (thymines on the opposite strand) within the target site are hypothesized to act in priming reverse transcription from the poly(A) tail of the LINE transcript. SINEs, which are mobilized by hijacking of the LINE machinery [17], have a similar target site preference as LINEs. While plant LINEs are predicted to move in a similar manner to mammalian LINEs, the consensus site has not yet been studied in a comprehensive manner. However, a study of *Arabidopsis *SINEs indicated a similar consensus sequence as mammalian LINEs; a string of adenines within the target site duplication, as well as a thymine at the 3' nicking site [18].

**Figure 4:**
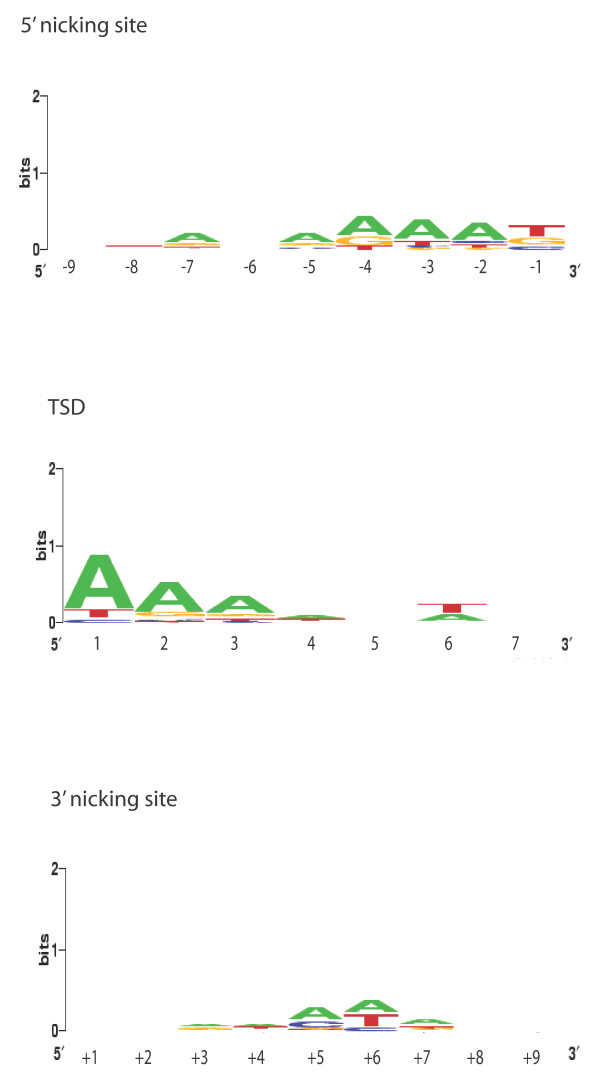
**Logo diagrams of consensus sequences at *Sadhu *insertion sites, based on 14 insertions in the Col reference genome**. Nine nucleotides proximal to the target site were examined as the 5' nicking site, while nine nucleotides distal to the target site were examined as the 3' nicking site. The first seven nucleotides within the target site duplication were examined.

A total of 14 *Sadhu *sequences containing target site duplications of between 7 and 16 bp were identified in the *A. thaliana *genome (Table 3). We examined the region around these target sites to determine whether 5' and 3' nicking site consensus patterns could be identified and, if so, whether they resembled patterns previously reported for LINEs and SINEs. As shown in Figure 4, the 5' nicking site does appear to favor a thymine (preceded by adenines), while the target site duplication also began with a stretch of adenines. There is no strong consensus at the 3' nicking site. These data are consistent with a model in which *Sadhu *elements, similar to SINEs, are mobilized by the LINE-encoded target primed reverse transcription machinery.

**Table 3:** Target site sequences of *Arabidopsis thaliana Sadhu *elements.

*Sadhu*	5' Nicking site	Target site duplication	3' Nicking site
1-1	tacaaaagt	aaatgactagtagga	taataaaca
1-2	acttgacat	agctatgaaaatcgt	tggaccatc
3-2	tttatgaag	aatcttcgtt	cagtcctgc
4-2	acaacattt	aaagatatctcgtttg	tggagaacg
5-1	ctgcaatat	agtactactact	aatgttatc
5-1d1	ttaagaaag	aaatgtgtctcaacg	cgaccaacg
6-1	gaagagacc	aaaacctagtctggag	tacaaagta
6-1d2	ttataaaag	aaaactaatcttaa	gaaaaatac
7-1	atggaagat	aaagaatctggcttt	ttgtaaaac
7-2	ctatggaag	aagaaggtaa	ccaactact
7L1	agggagttt	ttaagag	ttttattat
7L2	tcatataat	aattacctagca	cgaaatcta
8-1	gaacataac	aaaagatccaa	acgtatggt
9L3	caatcaacc	ccgtatt	gtagatttt

An examination of the *A. thaliana *Col reference genome [9] reveals less than 1,500 LINE superfamily-related elements spanning 12 different lineages, including both LINE1, LINE2, TA11 and TA12 families [19-21]. However, less than 50 LINEs in the *A. thaliana *reference genome are greater than 5,000 bp in length, and almost none contain intact open reading frames. Therefore, while it is evident that *Sadhu *elements have been mobile during the divergence of different *Arabidopsis *strains, their low copy number might be a consequence of the sheer rarity of active autonomous LINE driver elements.

### Sadhu elements can be identified in taxa outside of *A. thaliana*

In order to explore the evolutionary distribution of the *Sadhu *sequence family, we sought to identify *Sadhu *homologs in two related species of the *Brassicaceae *family, *A. arenosa *and *A. lyrata*. These species are estimated to have diverged from *A. thaliana *approximately 5 million years ago. The genomes of the three species have changed significantly in that interval: *Arabidopsis arenosa *and *Arabidopsis lyrata *maintain the ancestral complement of eight chromosomes, while *A. thaliana *has condensed its chromosome number to five [22,23]. Molecular evolutionary studies have determined that the average sequence divergence at silent sites between *A. thaliana *and *A. arenosa *or *A. lyrata *is 12% to 15% [22].

**Figure 5:**
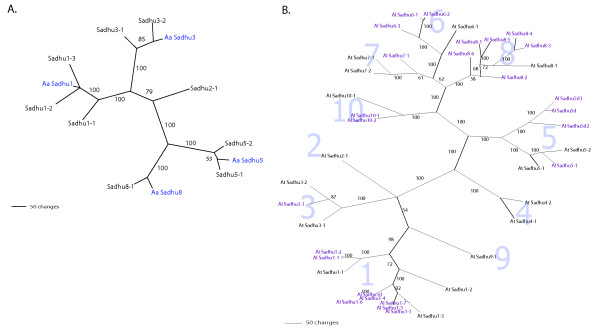
**Phylogenetic analysis of *Sadhu *sequences from *Arabidopsis arenosa *and *Arabidopsis lyrata *relative to *Arabidopsis thaliana***. (a) Maximum parsimony phylogram of *A. arenosa *(Aa) internal *Sadhu *sequence clones and related *A. thaliana *elements. *A. arenosa *sequences are in blue. (b) Maximum parsimony phylogram of *A. lyrata Sadhu *sequences >350 bp (Al, purple) and full-length *A. thaliana *elements. Shaded large numbers indicate *Sadhu *subfamilies. See to Additional file 4 for DNA sequences of *A. lyrata *elements. Bootstrap values (percentages) were calculated from 500 bootstrap replicates.

We attempted to isolate *Sadhu *elements from *A. arenosa*. DNA sequence was obtained from specific PCR products that were generated using *A. arenosa *genomic templates and primers corresponding to the *A. thaliana *elements *Sadhu5-1*, *Sadhu1-3*, *Sadhu3-1*, and *Sadhu8-1 *(Table 4; Additional file 2). In a phylogenetic analysis, the *A. arenosa Sadhu *sequences that we obtained cluster within the previously defined subfamilies (Figure 5a).

**Table 4:** *Sadhu *sequences from *Arabidopsis arenosa*.

Sequence name	Length (bp) of *Sadhu *sequence	Genbank accession number	*Arabidopsis thaliana *primer origin	Closest *A. thaliana *homolog (pairwise blast)	Percentage identity to *A. thaliana *ortholog
AaSadhu1	283	DQ680035	Sadhu1-3	Sadhu1-2	81
AaSadhu1FP1	204	EF535557	Sadhu1 5' TAIL	Sadhu1-1	74
AaSadhu1FP2	117	EF535558	Sadhu1 5' TAIL	Sadhu1-3	85
AaSadhu1FP3	45	EF535559	Sadhu1 5' TAIL	Sadhu1-3	82
AaSadhu1TP1	470	EF535560	Sadhu1 3' TAIL	Sadhu1-2	80
AaSadhu1TP2	232	EF535561	Sadhu1 3' TAIL	Sadhu1-3	84
AaSadhu1TP3	478	EF535565	Sadhu1 3' TAIL	Sadhu1-3	85
AaSadhu1TP4	480	EF535564	Sadhu1 3' TAIL	Sadhu1-3	84
AaSadhu3	686	DQ680038	Sadhu3-1	Sadhu3-2	86
AaSadhu3FP1	49	EF535567	Sadhu3 5' TAIL	Sadhu3-1	91
AaSadhu3TP1	188	EF535566	Sadhu3 3' TAIL	Sadhu3-2	86
AaSadhu5	344	DQ680036	Sadhu5-1, Sadhu5-2	Sadhu5-1	88
AaSadhu5FP1	94	EF535550	Sadhu5 5' TAIL	Sadhu5-1	87
AaSadhu5TP1	384	EF535551	Sadhu5 3' TAIL	Sadhu5-2	86
AaSadhu8	472	DQ680033	Sadhu8-1	Sadhu8-1	79
AaSadhu8FP1	202	EF535553	Sadhu8 5' TAIL	Sadhu8-1	76
AaSadhu8TP1	149	EF535556	Sadhu8 3' TAIL	Sadhu8-1	79

TAIL PCR = thermal asymmetric interlaced polymerase chain reaction.

We conducted TAIL PCR using *A. arenosa *genomic templates to identify more complete sequences for the *Sadhu *elements identified by PCR. Three 5' and four 3' flanking sequences homologous to *Sadhu1 *were amplified and cloned from *A. arenosa *genomic DNA template (Table 4, Additional file 3). Several of the 3' *Sadhu1 *portions were >95% identical to one another, indicative of recent retrotransposition in this subfamily. Two 5' flanking clones (*AaSadhu1FP3 *and *AlSadhu1FP1*) shared a stretch of 150 bp of sequence that does not correspond to known *Sadhu1 *sequence in *A. thaliana*. This extra sequence may have been transduced by the *Sadhu *element resulting in a chimeric retroposon.

Both 3' and 5' flanking sequences were obtained by TAIL PCR corresponding to *A. arenosa Sadhu3 *(Table 4 and Additional file 3). Because these sequences could not be joined by PCR, there are likely to be at least two members of this subfamily in *A. arenosa*. *Sadhu5 *TAIL PCR sequences isolated from *A. arenosa *were 85% to 88% identical to *A. thaliana Sadhu5 *subfamily members (5' and 3' portions) (Table 4 and Additional file 3). 5' and 3' sequences were also obtained corresponding to *Sadhu8 *subfamily members from *A. arenosa *(Table 4 and Additional file 3). These sequences were greater than 90% identical to one another and 75% to 79% identical to *A. thaliana Sadhu8-1*, indicating that retrotransposition occurred more recently than the divergence of the two species. In summary, *A. arenosa *contains several members of at least four *Sadhu *subfamilies. Examination of sequences flanking the *Sadhu *elements suggests that these elements are located in non-orthologous positions in *A. arenosa *relative to *A. thaliana *(Additional file 3).

*A. lyrata Sadhu *elements were identified from iterative BLAST searches of the recent *A. lyrata *genome sequence assembly (JGI V. 1.0; Joint Genome Institute, Walnut Creek, CA, USA). We used *A. thaliana *full-length *Sadhu *sequences as queries in a primary search to identify a set of *A. lyrata *sequences, which were subsequently used as queries in secondary searches. This method is expected to identify all full-length or near full-length sequences, although shorter *Sadhu*-related partial elements might have been overlooked. In total, we found 21 full-length and 4 partial *Sadhu *elements greater than 350 bp in length (Table 5, Additional file 4). The number of full-length elements (21) is similar to that in *A. thaliana *(16), indicating that the element family is relatively small in both species. Full-length *A. lyrata *elements are structurally similar to *Sadhu *elements in *A. thaliana*: they begin with a conserved motif (5' CAATCGTTSC 3' followed by a polypyrimidine patch) and terminate approximately 900 bp downstream in a poly(A) tract. Of the 21 full-length elements, 15 feature direct target site duplications of between 8 and 18 bp in length, suggesting that they originated via retrotransposition. There are no discernable conserved open reading frames. None of the elements appear in orthologous locations to *A. thaliana *elements, indicating that *Sadhu *elements have mobilized considerably since the divergence of the two species, and that related elements are similar through retrotransposition and not through direct inheritance of the genomic region.

**Table 5:** *Sadhu *elements >350 base pairs (bp) in the *Arabidopsis lyrata *genome.

Sequence name	JGI scaffold coordinates (approximate)	Orientation	Length (bp) of *Sadhu *sequence	Target site duplication (bp)	Full length?	Percentage identity to nearest *A. thaliana Sadhu*
AlSadhu1-1	7:7309496-7310418	-	948	18	Yes	86
AlSadhu1-2	8:11697563-11698467	+	922	12	Yes	86
AlSadhu1-3	6:22517373-22518173	+	957	14	Yes	84
AlSadhu1-4	3:1662010-1662618	-	1009	14	Yes	81
AlSadhu1-5	1:24954753-24955365	+	924	ND	Yes	84
AlSadhu1-6	4:841417-842023	+	965	16	Yes	82
AlSadhu1-6	3:13675822-13676434	+	927	16	Yes	84
AlSadhu1d	2:14298861-14299465	+	827	ND	No	81
AlSadhu3-1	7:12620425-12621361	+	928	18	Yes	86
AlSadhu5-1	7:17697122-17698008	+	879	11	Yes	85
AlSadhu5d	6:5062639-5062768	-	791	ND	No	72
AlSadhu5d2	6:25041036-25041746	+	804	ND	No	73
AlSadhu5d3	5:4156620-4157046	-	395	ND	No	71
AlSadhu6-1	3:21898960-21899646	-	899	14	Yes	77
AlSadhu6-2	2:14183205-14186662	+	887*	15	Yes	77
AlSadhu6-3	8:5795744-5796493	+	927	ND	Yes	78
AlSadhu7-1	7:4360460-4360769	-	901	16	Yes	79
AlSadhu8-1	1:13276396-13277158	-	920	13	Yes	77
AlSadhu8-2	3:807549-808169	+	865	ND	Yes	79
AlSadhu8-3	2:25942-26642	+	908	8	Yes	77
AlSadhu8-4	8:14602238-14602861	+	875	15	Yes	75
AlSadhu8-5	7:17882685-17884241	+	930**	ND	Yes	78
AlSadhu8-6	6:17227119-17227908	-	910	ND	Yes	77
AlSadhu10-1	3:4473616-4474232	+	918	17	Yes	80
AlSadhu10-2	2:9675105-9675721	+	895	ND	Yes	80

*interrupted by 2,028 nt non-*Sadhu *sequence; **Interrupted by 518 nt non-*Sadhu *sequence; bp = base pairs; JGI = Joint Genome Institute; ND = not detected; nt = nucleotides.

*A. lyrata *elements are between 71% and 86% identical to the most similar *A. thaliana *element (Table 5). Figure 5b shows a phylogenetic tree showing the relationships among the 25 *A. lyrata *and 16 full-length *A. thaliana *elements. All *A. lyrata *elements clustered within previously defined subfamilies, indicating that the divergence of the different subfamilies predated the split of these two species. Most of the *Sadhu *subfamilies previously identified in *A. thaliana *have representatives in *A. lyrata*; however, there is a dramatic expansion of elements within certain subfamilies relative to others (Figure 5b, Table 5). For instance, the *Sadhu1 *subfamily contains three members in *A. thaliana *but has expanded to seven full-length members in *A. lyrata*. The *Sadhu8 *and *Sadhu6 *subfamilies are represented by only a single member in *A. thaliana*, but feature six and three full-length elements, respectively, in *A. lyrata*. These genome comparisons suggest that, while multiple distinct *Sadhu *subfamilies have been active since the divergence of these two taxa, different subfamilies have proliferated more in certain species than in others. Alternatively, certain subfamilies may have been pared down by deletion and elimination in one species relative to the other.

### Perspective

We have identified *Sadhu *sequences corresponding to multiple subfamilies in the related species *A. lyrata *and *A. arenosa*. The presence of target site duplications and poly(A) tracts, along with the absence of orthologous sites, strongly suggests that *Sadhu *elements in these other taxa arose via retrotransposition. In a few cases, elements within a given species are greater than 95% identical to one another, indicating that these sequences have mobilized more recently than the divergence of the different species. The partial sequence available for the *Brassica *genome [24] does not contain *Sadhu-*related sequences. While these sequences may have been lost from some taxa, the high degree of divergence amongst elements in the *Arabidopsis *genus strongly suggests an ancient origin for these elements. Therefore, we predict that some sequences related to *Sadhu *elements might be present in other plants, perhaps even those quite distantly related to *Arabidopsis*. These presumably more divergent *Sadhu *relatives might share little overall primary nucleotide sequence with the *A. thaliana *elements, but might have maintained other recognizable diagnostic features, such as length, conserved 5' motif(s), a 3' poly (A) tract, and target site duplications.

Low copy number and high divergence among element subfamilies is not a phenomenon unique to *Sadhu *elements. Indeed, because only 10% of the *Arabidopsis *genome is composed of transposable elements [25], lower than other sequenced plant genomes, there may be a general tendency for genome size reduction in this species through progressive loss of repetitive DNA. A comparison of the *A. thaliana *genome with the five times larger *Brassica oleracea *genome revealed that while most element families were present in both species, some (for example, *CACTA *elements) had contributed more than others to the relative expansion of the *Brassica *genome [21]. As with the different *Sadhu *subfamilies, different SINE non-LTR subfamilies appear to be more active in each of the two species [26]. The lack of orthologous *Sadhu *insertion sites among different *Arabidopsis *species is also reminiscent of the case with SINEs, which similarly featured no shared sites in *B. oleracea *[26]. Both types of non-LTR elements are therefore subject to frequent loss over evolutionary time. This susceptibility may be a consequence of the dispersed pattern of localization of *Sadhus *and SINEs: elements that target heterochromatic regions, such as *Athila *LTR elements, appear to be relatively protected from this winnowing process [27].

Although retroelement superfamilies can typically be found in widely differing plant taxa [8], certain families show longer phylogenetic branch lengths and low copy numbers more similar to the case with *Sadhu*. In particular, *copia/Ty1 *families in *Arabidopsis *are highly divergent from one another [19,28-30]. Non-LTR TA elements are also present in few copies per genome from distinct, evolutionarily ancient lineages [20]. This high divergence among element subfamilies and lack of orthologous sites in related species stands in stark contrast to primate non-LTR elements: L1s and Alus crowd mammalian genomes, with both currently active lineages as well as many defunct ancestral sites shared among humans and their most recent relatives (for example, [31-33]). Therefore, while the evolutionary trajectory of *Sadhu *elements is not dramatically different from that exhibited by some plant retroelements, it is unlike many more well-studied elements.

## Conclusions

*Sadhu *elements represent a previously little characterized retrotransposon family. We have generated a comprehensive classification scheme for these sequences based on phylogenetic analysis. Partial elements often contain 3' poly(A) tracts and target site duplications, consistent with an origin by target primed reverse transcription-driven retrotransposition. An examination of the *Sadhu5 *subfamily among different *A. thaliana *strains indicates that subfamily members arose through retrotransposition; the presence of polymorphic insertion sites provides evidence for retrotransposition in the recent history of the species. In addition, sequences at the target site are similar to the *Arabidopsis *SINE consensus, consistent with the hypothesis that the LINE machinery is responsible for the mobilization of both of these types of elements. *Sadhu*-related sequences identified in *A. lyrata *and *A. arenosa *cluster within specific *A. thaliana *subfamilies, indicating that the radiation of this element family preceded the divergence of the *Arabidopsis *genus. These *A. lyrata *and *A. arenosa *elements often contain poly(A) tracts and target site duplications, consistent with the model that these sequences also arose via retrotransposition. Taken together, these studies indicate that *Sadhu *elements have been active since the divergence of different *Arabidopsis *species, and through the differentiation of different *A. thaliana *strains. Further research is warranted to resolve the molecular origin and potential impact of this unique class of DNA sequence on genome structure and organization.

## Methods

### Plant materials

*A. thaliana *strains were obtained from the *Arabidopsis *Biological Resource Center (ABRC, Columbus, OH, USA). Stock numbers are listed in Table 2. *A. arenosa *seeds were obtained from Craig Pikaard (Department of Biology, Indiana University, Bloomington, IN, USA). Plants were grown on soil or on 1 × MS media with 1% sucrose. DNA was isolated using previously described methods [34].

### Molecular biology

PCR was performed using standard conditions with *Taq *DNA polymerase (QIAGEN, Valencia, CA, USA) or KT1 polymerase (Clontech, Mountain View, CA, USA). Two rounds of TAIL PCR were performed on *A. arenosa *template using protocols and degenerate AD primers described previously [35]. Products from the second round of TAIL PCR were isolated from agarose gel and TA cloned into pGEM-T Easy (Promega, Madison, WI, USA) before sequencing. All other PCR products were directly sequenced without an additional cloning step following purification through Performa DTR gel filtration cartridges (Edge BioSystems, Gaithersburg, MD, USA). DNA sequencing was performed using Big Dye Terminator Cycle Sequencing (PerkinElmer, Waltham, MA,, USA) protocols/reagents; sequences were processed at the Washington University Department of Biology sequencing facility. PCR primers used to generate the data in Tables 2 and 4 are described in Additional file 2. 'Internal' PCR primers were used to amplify sequence from different *A. thaliana *strains and to amplify homologs from *A. arenosa*. All sequences in this study have been deposited in the National Center for Biotechnology Information (NCBI) database. Genbank accession numbers are listed in Table 3 (for *A. arenosa *sequences) and in the legend to Figure 3 (for *A. thaliana *strain specific sequences).

### Computational analysis

Full-length and partial *Sadhu *elements were identified based on sequence similarity to *At2 g01410 *as previously described [1]. The maximum parsimony and neighbor joining trees in Figures 1 and 5 were generated using the software PAUP* V. 4.0 (Sinauer Associates, Sunderland, MA, USA) based on a ClustalX alignment [36]. Divergence matrices in Additional file 1 were generated based on a ClustalX alignment using the European Molecular Biology Open Software Suite (EMBOSS) program 'distmat' [37] run without corrections. Consensus sequences of different subfamilies were generated from full-length and derivative sequences using the EMBOSS program 'cons' [37]. Alignments in Figure 3 were visualized by ClustalX [36]. WebLogo [38] was used to create the logo images in Figure 4 that describe the retrotransposition target consensus sites. Annotations of features within TAIL PCR products in Additional file 3 were aided by the repeat masker feature on the Censor server [39] and the TAIR WU-BLAST server [40]. *A. lyrata *sequence information was obtained using the database, browser, and BLAST tools at the Joint Genome Institute (JGI) [41]. *A. lyrata Sadhu *elements were identified by iterative BLAST searches of the JGI assembly using, initially, *A. thaliana *and then *A. lyrata Sadhu *sequences as queries until a self-referencing set of sequences was identified. The classification scheme in Table 1 and locus ID and nucleotide positions for full-length elements have been submitted to both The *Arabidopsis *Information Resource (TAIR) [9] as well as the repeat database at the Genetic Information Research Institute (GIRI) [42].

## Abbreviations

BLAST: basic local alignment search tool; GIRI: Genetic Information Research Institute; JGI: Joint Genome Institute; LINE: long interspersed nuclear element; LTR: long terminal repeat; SINE: short interspersed nuclear element; TAIL PCR: thermal asymmetric interlaced polymerase chain reaction; TAIR: The *Arabidopsis *Information Resource; TSD: target site duplication.

## Competing interests

The authors declare that they have no competing interests.

## Authors' contributions

SHR designed and performed all experiments, conducted analysis and drafted the manuscript. EJR conducted analysis and revised and approved the manuscript.

## Supplementary Material

Additional file 1 **Divergence matrices of *Arabidopsis thaliana Sadhu *elements**. Additional file 1 is a spreadsheet file containing divergence matrices of *A. thaliana Sadhu *elements, both within subfamilies and of consensus sequences across subfamilies. These matrices are based on ClustalX multiple sequence alignment. Click here for file

Additional file 2 **Polymerase chain reaction (PCR) primers**. Additional file 2 is a table listing PCR primers used in this study. Click here for file

Additional file 3 **DNA sequence information for *Sadhu *sequences greater than 350 base pairs (bp) in the *Arabidopsis lyrata *genome assembly**. Additional file 4 provides DNA sequence information for *Sadhu *sequences greater than 350 bp in the *Arabidopsis lyrata *genome assembly. Target site duplications are indicated in purple and the conserved CAATCGTTSC motif is italicized and underlined. Non-*Sadhu *sequence inserted in the elements is in gray and italicized Click here for file

Additional file 4 **Partial *Sadhu *elements and flanking genomic sequences identified in *Arabidopsis arenosa***. Additional file 3 contains diagrams of partial *Sadhu *elements and flanking genomic sequences identified in *A. arenosa*. (a) *Sadhu1*; (b) *Sadhu3*; (c) *Sadhu5*; (d) *Sadhu8*. The scale is indicated. Internal polymerase chain reaction (PCR) sequences used specific primers based on the *Arabidopsis thaliana *sequence, while 5' and 3' sequences were obtained by thermal asymmetric interlaced (TAIL) PCR (see Table 4 for details). 5' *Sadhu *sequences are in blue, 3' *Sadhu *sequences are orange. Gray dotted arrows indicate the extent of *Sadhu *sequence homology. Features in flanking sequences are marked as green boxes. The inverted arrow in the annotation of the Aa5FP1 clone indicates the direction of transcription of the flanking gene-related sequence. *Sadhu5 *and *Sadhu8 *3' sequences feature poly(A) tracts at the *Sadhu *boundary, consistent with retrotransposition. Click here for file

## References

[B1] Rangwala SH Elumalai R Vanier C Ozkan H Galbraith DW Richards EJ Meiotically stable natural epialleles of *Sadhu*, a novel *Arabidopsis *retroposon PLoS Genet 2006 2 e36 10.1371/journal.pgen.0020036 16552445 PMC1401498

[B2] Weiner AM SINEs and LINEs: the art of biting the hand that feeds you Curr Opin Cell Biol 2002 14 343 350 10.1016/S0955-0674(02)00338-1 12067657

[B3] Santi L Wang Y Stile MR Berendzen K Wanke D Roig C Pozzi C Müller K Müller J Rohde W Salamini F The GA octodinucleotide repeat binding factor BBR participates in the transcriptional regulation of the homeobox gene Bkn3 Plant J 2003 34 813 826 10.1046/j.1365-313X.2003.01767.x 12795701

[B4] Sangwan I O'Brian MR Identification of a soybean protein that interacts with GAGA element dinucleotide repeat DNA Plant Physiol 2002 129 1788 1794 10.1104/pp.002618 12177492 PMC166767

[B5] Granok H Leibovitch BA Shaffer CD Elgin SC Chromatin. Ga-ga over GAGA factor Curr Biol 1995 5 238 241 10.1016/S0960-9822(95)00048-0 7780729

[B6] Rangwala SH Richards EJ Differential epigenetics regulation within an *Arabidopsis *retroposon family Genetics 2007 176 151 160 10.1534/genetics.107.071092 17339215 PMC1893068

[B7] Evgen'ev MB Arkhipova IR Penelope-like elements - a new class of retroelements: distribution, function and possible evolutionary significance Cytogenet Genome Res 2005 110 510 521 10.1159/000084984 16093704

[B8] Voytas DF Cummings MP Koniczny A Ausubel FM Rodermel SR copia-like retrotransposons are ubiquitous among plants Proc Natl Acad Sci USA 1992 89 7124 7128 10.1073/pnas.89.15.7124 1379734 PMC49658

[B9] Swarbreck D Wilks C Lamesch P Berardini TZ Garcia-Hernandez M Foerster H Li D Meyer T Muller R Ploetz L Radenbaugh A Singh S Swing V Tissier C Zhang P Huala E The *Arabidopsis *Information Resource (TAIR): gene structure and function annotation Nucleic Acids Res 2008 36 D1009 1014 10.1093/nar/gkm965 17986450 PMC2238962

[B10] Bibillo A Eickbush TH End-to-end template jumping by the reverse transcriptase encoded by the R2 retrotransposon J Biol Chem 2004 279 14945 14953 10.1074/jbc.M310450200 14752111

[B11] Buzdin AA Retroelements and formation of chimeric retrogenes Cell Mol Life Sci 2004 61 2046 2059 10.1007/s00018-004-4041-z 15316654 PMC11138840

[B12] Ziolkowski PA Koczyk G Galganski L Sadowski J Genome sequence comparison of Col and Ler lines reveals the dynamic nature of *Arabidopsis *chromosomes Nucleic Acids Res 2009 37 3189 3201 10.1093/nar/gkp183 19305000 PMC2691826

[B13] Luan DD Korman MH Jakubczak JL Eickbush TH Reverse transcription of R2Bm RNA is primed by a nick at the chromosomal target site: a mechanism for non-LTR retrotransposition Cell 1993 72 595 605 10.1016/0092-8674(93)90078-5 7679954

[B14] Feng Q Moran JV Kazazian HH Jr Boeke JD Human L1 retrotransposon encodes a conserved endonuclease required for retrotransposition Cell 1996 87 905 916 10.1016/S0092-8674(00)81997-2 8945517

[B15] Jurka J Sequence patterns indicate an enzymatic involvement in integration of mammalian retroposons Proc Natl Acad Sci USA 1997 94 1872 1877 10.1073/pnas.94.5.1872 9050872 PMC20010

[B16] Szak ST Pickeral OK Makalowski W Boguski MS Landsman D Boeke JD Molecular archeology of L1 insertions in the human genome Genome Biol 2002 3 0052 10.1186/gb-2002-3-10-research0052 PMC134481 12372140

[B17] Dewannieux M Heidmann T LINEs, SINEs and processed pseudogenes: parasitic strategies for genome modeling Cytogenet Genome Res 2005 110 35 48 10.1159/000084936 16093656

[B18] Myouga F Tsuchimoto S Noma K Ohtsubo H Ohtsubo E Identification and structural analysis of SINE elements in the *Arabidopsis thaliana *genome Genes Genet Syst 2001 76 169 179 10.1266/ggs.76.169 11569500

[B19] Kapitonov VV Jurka J Molecular paleontology of transposable elements from *Arabidopsis thaliana* Genetica 1999 107 27 37 10.1023/A:1004030922447 10952195

[B20] Wright DA Ke N Smalle J Hauge BM Goodman HM Voytas DF Multiple non-LTR retrotransposons in the genome of *Arabidopsis thaliana* Genetics 1996 142 569 578 8852854 10.1093/genetics/142.2.569 PMC1206989

[B21] Zhang X Wessler SR Genome-wide comparative analysis of the transposable elements in the related species *Arabidopsis thaliana *and *Brassica oleracea* Proc Natl Acad Sci USA 2004 101 5589 5594 10.1073/pnas.0401243101 15064405 PMC397431

[B22] Clauss MJ Koch MA Poorly known relatives of *Arabidopsis thaliana* Trends Plant Sci 2006 11 449 459 10.1016/j.tplants.2006.07.005 16893672

[B23] Koch MA Matschinger M Evolution and genetic differentiation among relatives of *Arabidopsis thaliana* Proc Natl Acad Sci USA 2007 104 6272 6277 10.1073/pnas.0701338104 17404224 PMC1851049

[B24] *Brassica *sequence http://brassica.bbsrc.ac.uk/

[B25] Arabidopsis Genome Initiative Analysis of the genome sequence of the flowering plant *Arabidopsis thaliana* Nature 2000 408 796 815 10.1038/35048692 11130711

[B26] Lenoir A Pelissier T Bousquet-Antonelli C Deragon JM Comparative evolution history of SINEs in *Arabidopsis thaliana *and *Brassica oleracea*: evidence for a high rate of SINE loss Cytogenet Genome Res 2005 110 441 447 10.1159/000084976 16093696

[B27] Pereira V Insertion bias and purifying selection of retrotransposons in the *Arabidopsis thaliana *genome Genome Biol 2004 5 R79 10.1186/gb-2004-5-10-r79 15461797 PMC545599

[B28] Konieczny A Voytas DF Cummings MP Ausubel FM A superfamily of *Arabidopsis thaliana *retrotransposons Genetics 1991 127 801 809 1709409 10.1093/genetics/127.4.801 PMC1204407

[B29] Terol J Castillo MC Bargues M Perez-Alonso M de Frutos R Structural and evolutionary analysis of the copia-like elements in the *Arabidopsis thaliana *genome Mol Biol Evol 2001 18 882 892 11319272 10.1093/oxfordjournals.molbev.a003870

[B30] Voytas DF Konieczny A Cummings MP Ausubel FM The structure, distribution and evolution of the Ta1 retrotransposable element family of *Arabidopsis thaliana* Genetics 1990 126 713 721 2174394 10.1093/genetics/126.3.713 PMC1204225

[B31] Chimpanzee Sequencing and Analysis Consortium Initial sequence of the chimpanzee genome and comparison with the human genome Nature 2005 437 69 87 10.1038/nature04072 16136131

[B32] Lee J Cordaux R Han K Wang J Hedges DJ Liang P Batzer MA Different evolutionary fates of recently integrated human and chimpanzee LINE-1 retrotransposons Gene 2007 390 18 27 10.1016/j.gene.2006.08.029 17055192 PMC1847406

[B33] Liu GE Alkan C Jiang L Zhao S Eichler EE Comparative analysis of Alu repeats in primate genomes Genome Res 2009 19 876 885 10.1101/gr.083972.108 19411604 PMC2675976

[B34] Cocciolone SM Cone KC Pl-Bh, an anthocyanin regulatory gene of maize that leads to variegated pigmentation Genetics 1993 135 575 588 7694886 10.1093/genetics/135.2.575 PMC1205657

[B35] Liu YG Mitsukawa N Oosumi T Whittier RF Efficient isolation and mapping of *Arabidopsis thaliana *T-DNA insert junctions by thermal asymmetric interlaced PCR Plant J 1995 8 457 463 10.1046/j.1365-313X.1995.08030457.x 7550382

[B36] Thompson JD Gibson TJ Plewniak F Jeanmougin F Higgins DG The CLUSTAL_X windows interface: flexible strategies for multiple sequence alignment aided by quality analysis tools Nucleic Acids Res 1997 25 4876 4882 10.1093/nar/25.24.4876 9396791 PMC147148

[B37] Rice P Longden I Bleasby A EMBOSS: the European Molecular Biology Open Software Suite Trends Genet 2000 16 276 277 10.1016/S0168-9525(00)02024-2 10827456

[B38] Crooks GE Hon G Chandonia JM Brenner SE WebLogo: a sequence logo generator Genome Res 2004 14 1188 1190 10.1101/gr.849004 15173120 PMC419797

[B39] Censor server http://www.girinst.org/censor/

[B40] TAIR WU-BLAST server http://www.arabidopsis.org/wublast/index2.jsp

[B41] Joint Genome Institute BLAST tools http://genome.jgi-psf.org/Araly1/Araly1.home.html

[B42] Jurka J Kapitonov VV Pavlicek A Klonowski P Kohany O Walichiewicz J Repbase Update, a database of eukaryotic repetitive elements Cytogenet Genome Res 2005 110 462 467 10.1159/000084979 16093699

