# Supplementary material for: The structure, organization and radiation of Sadhu non-long terminal repeat retroelements in Arabidopsis species
Source: Mob DNA. 2010 Mar 1;1:10. doi: 10.1186/1759-8753-1-10 (PMC2848041; doi:10.1186/1759-8753-1-10)
Supplement: Additional file 2 — Polymerase chain reaction (PCR) primers. Additional file 2 is a table listing PCR primers used in this study. [file 1759-8753-1-10-S2.DOC]

| PCR primers used in this study | | |
| --- | --- | --- |
| Primer name | Sequence (5’ -> 3’) | Use |
| 4g01525F1 | CCT CTC TTC TCT CTC CTC ACT G | Sadhu5-1 internal |
| 4g01525R1 | TAG AAC CCA CAA AAT TGG GCT GG | Sadhu5-1 internal |
| 4g01525R4 | CAG TGA GGA GAG AGA AGA GAG G | Sadhu5-1 5’ flanking |
| 4g01525F4 | GTA AAG CCT CCA AAG TCA GCC TC | Sadhu5-1 5’ flanking |
| 4g01525F41 | CAC TTC CTC TCC CGT CGG AC | Sadhu5-1 5’ flanking |
| 4g01525R3 | GAA GTG GTG TGA CTC TTC TCT AGT TC | Sadhu5-1 3’ flanking |
| 4g01525F3 | CCC AGC CCA ATT TTG TGG GTT C | Sadhu5-1 3’ flanking |
| 4g01525R31 | TAT CTA CCT TTT AGC ACC CGA GC | Sadhu5-1 3’ flanking |
| 4g01525F2 | ATT AGC GCA TCC ACA TCC GGC ATC | Sadhu5-1 5’ empty site |
| 4g01525ES | TAG ATA CGG TTC GTT AGA ATT GT | Sadhu5-1 3’ empty site |
| 1g18190F1 | CTC AAC GAT GAA AAG TCC GAG G | Sadhu5-1d1 internal |
| 1g18190R1 | AAC CTA CAA AAT TGG GTT GGG C | Sadhu5-1d1 internal |
| 1g18190F3 | TGA GCC AAT CCC GTG GAT TAA AAC | Sadhu5-1d1 5’ flanking |
| 1g18190R3 | CCT CGG ACT TTT CAT CGT TGA G | Sadhu5-1d1 5’ flanking |
| 1g18190F31 | AGC TTC GAC GAG ATC CAA TTC G | Sadhu5-1d1 5’ flanking |
| 1g18190F2 | GCC CAA CCC AAT TTT GTA GGT TC | Sadhu5-1d1 3’ flanking |
| 1g18190R2 | GAA GGA TAT CTT AGC CTT TGC CG | Sadhu5-1d1 3’ flanking |
| 1g18190R21 | AGG GAT GAG GAA ACA GAA TTG CG | Sadhu5-1d1 3’ flanking |
| 1g18190ES1 | CCT CTC TTA TGA TGT GGG ATG GAG | Sadhu5-1d1 5’ empty site |
| 1g18190ES2 | TTA GGC ATC ATC GCC ACA GCA G | Sadhu5-1d1 3’ empty site |
| 4g00955F1 | GGA TCC ATC TCA AAC TTT CTT CCA C | Sadhu5-1d2 internal |
| 4g00955R1 | CCT ACA CAA TTG GAC TGG GG | Sadhu5-1d2 internal |
| 4g00955F2 | GTA CGG TTC AAG GAC AAG ACT ACT G | Sadhu5-1d2 5’ flanking |
| 4g00955R2 | GTG GAA GAA AGT TTG AGA TGG ATC C | Sadhu5-1d2 5’ flanking |
| 4g00955F3 | CCC CAG TCC AAT TGT GTA GG | Sadhu5-1d2 3’ flanking |
| 4g00955R3 | GCA AAT GCA CAT GTC TCC AAT TGC | Sadhu5-1d2 3’ flanking |
| 5g27927F1 | GGT GAA CAA GAT CCC TTC TCC G | Sadhu5-2 internal |
| 5g27927R1 | CCT CCT TTT CAA TCC GGC GAA TC | Sadhu5-2 internal |
| 5g27927F2 | CAC AAC ATC ATC ATG CCA TGA CG | Sadhu5-2 5’ flanking |
| 5g27927R2 | CGG AGA AGG GAT CTT GTT CAC C | Sadhu5-2 5’ flanking |
| 5g27927F21 | GGA TGT GAT CTC TAA CCA AGA GGC | Sadhu5-2 5’ flanking |
| 5g27927F3 | GAT TCG CCG GAT TGA AAA GGA GG | Sadhu5-2 3’ flanking |
| 5g27927R1 | GCC CTG AAG TTC ACT CTT CAT CG | Sadhu5-2 3’ flanking |
| 5g27927R31 | CCA CGT CCG TGC TTA GCA CG | Sadhu5-2 3’ flanking |
| 5g28626R1 | AAT CCA TCA ACT ACC GAG ATC CG | Sadhu1-3 internal |
| 5g28626F1 | CTT GAT GGG TTG GAG GGT GAA AC | Sadhu1-3 internal |
| 3g44040F3 | CAA TCG TTC CAC GTC TCT CTC TTC | Sadhu3-1 internal |
| 3g44040R1 | GAC GCG AGA TCG GAT TTG GAG | Sadhu3-1 internal |
| 1g50735F1 | TTG GCG GAG GAG GAG CAT TG | Sadhu8-1 internal |
| 1g50735R1 | CGT TCG CAG AGT GAC CAA ATC | Sadhu8-1 internal |
| Sadhu5TR2 | GCA ACC ACA TCG AAG GGG AG | Sadhu5 5’ TAIL PCR from A. arenosa |
| Sadhu5TR1 | AGC GAA TCT CTA CTT CCG ACA CG | Sadhu5 5’ TAIL PCR from A. arenosa |
| Sadhu5TL2 | GGC CGG ATT GAA GGT TTT TGG | Sadhu5 3’ TAIL PCR from A. arenosa |
| Sadhu5TL1 | GCGACCTGCTTCCTCCTG | Sadhu5 3’ TAIL PCR from A. arenosa |
| Sadhu8TR2 | TCA CTG CAT CAA TGC TCC TCC | Sadhu8 5’ TAIL PCR from A. arenosa |
| Sadhu8TR1 | GGA GCA TCG ATA CAA CTC CC | Sadhu8 5’ TAIL PCR from A. arenosa |
| Sadhu8TL2 | CTT CCT CCA GTT TTC TCA ATC CTC | Sadhu8 3’ TAIL PCR from A. arenosa |
| Sadhu8TL1 | GGT TGG TGC GAC GGA AGA G | Sadhu8 3’ TAIL PCR from A. arenosa |
| Sadhu1TR2 | CCG TGA AGG AGA GCC ACC | Sadhu1 5’ TAIL PCR from A. arenosa |
| Sadhu1TR1 | AGA TCC AAT CCA CCG GAG TC | Sadhu1 5’ TAIL PCR from A. arenosa |
| Sadhu1TL2 | GGG TTC GCC ACT TTG CGA AG | Sadhu1 3’ TAIL PCR from A. arenosa |
| Sadhu1TL1 | GCG GCG GAA GTT GAG TG | Sadhu1 3’ TAIL PCR from A. arenosa |
| Sadhu3RL2 | AAG CAG TGC TTC TCG TAA TCG C | Sadhu3 5’ TAIL PCR from A. arenosa |
| Sadhu3RL1 | CAG GTT TGT CGT CGG GAT CG | Sadhu3 5’ TAIL PCR from A. arenosa |
| Sadhu3TL2 | GAG AGG AAA GCT CGC TCA CC | Sadhu3 3’ TAIL PCR from A. arenosa |
| Sadhu3TL1 | GCC ACA GAT GCA TCA CGA ACC | Sadhu3 3’ TAIL PCR from A. arenosa |

Unless otherwise indicated, PCR primers were designed against *A. thaliana* Col reference genome sequence. “Internal” PCR primers were also used to amplify homologous elements from *A. arenosa*.
