# Supplementary material for: The structure, organization and radiation of Sadhu non-long terminal repeat retroelements in Arabidopsis species
Source: Mob DNA. 2010 Mar 1;1:10. doi: 10.1186/1759-8753-1-10 (PMC2848041; doi:10.1186/1759-8753-1-10)
Supplement: Additional file 3 — DNA sequence information for Sadhu sequences greater than 350 base pairs (bp) in the Arabidopsis lyrata genome assembly. Additional file 4 provides DNA sequence information for Sadhu sequences greater than 350 bp in the Arabidopsis lyrata genome assembly. Target site duplications are indicated in purple and the conserved CAATCGTTSC motif is italicized and underlined. Non-Sadhu sequence inserted in the elements is in gray and italicized [file 1759-8753-1-10-S3.DOC]

**Additional Data File 3.** *A. lyrata Sadhu* elements.

>AlSadhu1-1 Araly1 scaffold_7: 7309496-7310418 strand=-

AAAAGAAAATGCATAAGAGAATATTACAACAATATAAAAATCGGA*CAATCGTTCC*TCTCTCTTCTCTCTCTTCTCTCTCTTCCTTTTCTAGATCTAGGATTTTCTCGCGGCCGCCTTTGTTAAAGAGCTTCATCGACGGAGGTTGGGTTAGCGGCTGAAGCGGTGGTGGGTGCCTCTCTTCTCCGGTGGTGTGTGGTGTGGTGGATCTGTGGTTTCGATGCAAATCGGTGGCTCTCCTCACGGCAGCTATAAGATGATGTTCCTCCGTGTCTGCTGGTTAAAGGACGCCGACGGATTGGAATAGATTCTTTAGGTTCGGCGGCTTGTGAGTTGTAGCTCCGGTGAGCGGCGGAAGTCGGACTATGGATCGGTGGGTTCACCACTTTGCGAAGGGATTAACGGTGGTTCTTTGGCCGGATCCTAGATGGCGAGCTGCGGGTGTTCCTTGGCCGGATCTTGGTGGAAGATGGTTCGTCTGAGCCAACGACTCTCTTCCGCCGCCATGGCTGGTTTTTCCTTCGCCGGATCTGAAGTAAGGTTTGCTCTAGGGGAGGTTTGAGGGTGGGCATCGGTGGAGGTGAGGGTGAGTTTCGATTCCTTCTTCTTGCTAAGTTGGGGAGATGAGGTATTGGGGCCCGGTTGTGGCGGTTTTCCCTTTTCCGAAAGGAATGTTCCAGGTTTGGCTTTTTCTCAGCGATGGTTTGCGGCGGGGTTCGCTAGCTCAATCCAAAAACTTGATGTCGAGGTTGTAGCCACAAAGGAGCCGTCCTTGCTAGCGTCGGATCTAGAAGGCGAAGTTCGTGTTTTCCGGTGTATTGCACGGAGCTGATCTTGTTTTCGCTGGTTTTCGTTTTGGGCCTCTTCTATTTTGTAACCGTGGTTGGTCGTTTAGCCCAACTTTCATAAGCCCATTTGGGATTAATAAATTAAATTTTAAAATTAAAAAAAAAAAAAAAAAAAAAAAAAAAAAGAAAATGCATAAGA

>AlSadhu1-2 Araly1 scaffold_8: 11697563-11698467 strand=+

AAGATACAGTAAATAATATTACAACAATATAAAAATCGGA*CAATCGTTCC*TCTCTCTTCTCTCTCTTCCTTTCCTAGATCTAGGGTTTTCTCGCGGCTGCCTTTGTTAAAGAGCTTCATCGACGGAGGCTGGGTTAGCGGCTGAAGCGGTGGTGGGTGCCTCTCTTCTCCGGTGGTGTGTGGTGTGGTGGATCTGTGGTTTCGATGCAAATCGGTGGCTCTCCTCACGGCAGCTATAAGATGATGTTCCTCCGTGTCTGCTGGTTAAAGGACGCCGGCGGATTGGAATAGATTCTTTAGGTTCGGCGGCTTGTGAGTTGTAGCTCCGGTGAGCGGCGGAAGTCGGACTATGGATCGGTGGGTTCACCACTTTGCGAAGGGATTAACGGTGGTTCTTTGTCCGGATCCTAGATGGTGAGCTGCGGGTGTTCCTTGGCCGGATCTTGGTGGAAGATGGTTCGTCTGAGCCAACGACTCTTCCGCCGCCGTGGCTGGTTTTTCCTTCGCCGGATCTGAAGTAAGGTTTGCTCTAGGGGAGGTTTGAGGGTGGGCATCGGTGGAGGTGAGGGTGAGTTTCGATTCCTTCTTCTTGCTAGGTTGGGGAGATGAGGTATTGGGGCCCGGTTGTGGCGGTTTTCCCTTTTCCGAAAGGAATGTTCCAGGTTTGGCTTTTTCTCAGCGATGGTTTGCGGCGGGGTTCGCTAGCTCAAGCCAAAAACTTGATGTCGAGGTTGTAGCCACAAAGGAGCCGTCCTTGCTAGCGTCGGATCTAGAAGGCGAAGTTCGTGTTTTCCGGTGTACTGCACGGAGCTGATCTTGTTTTCGCTGGTTTTCGTTTTGGGCCTCTTCTATTTTGTAACCGTGGTTGGTCGTTTAGCTCAATTTTCATAAGTCCATTTGGGATTAATAAATTAAATTAAAAAAAAAAAAAAAAAAAGATACAGTAA

>AlSadhu1-3 Araly1 scaffold_6: 22517373-22518173 strand=+
CAATAATGAATATAAATCTTGTGACGAAAAAAAGGGA*CAATCGTTCC*CATTGAAACCCTCTCTTCTCTCTCCTAAAGCCAGATCTTAGATCTACTTCATCTCCTTTTTTCTTTTTTCGTTTATTGATCTTCAGTGTCGGTTGATGGGTGAGGGAGTGAAGCGGTGGTGAGCGTCTTTCTTCTCCGGTGGTGTGAGGTGTGGTTTGGTCTTTGGCTCATTGCTTGTTGGTGGCTCTCCTTCACGGCGGCCAGAGAAGCGGTTCCTTCGTTCTGCTGGTTAAGGACAACAGTAGATTGGATCTTGTTTCTCACGGCTCGGCGGCTTGTGATTGTGGCTCCGGCGAGTAGCGGAAGTTGAGCTTTCGATCGGTGGGTTTCACCACTTTGCGAAGAGGTTAACGGTGGCTCCTTGGCAGGATCCTAGATGGCTAGTTTCGGGTGATCCTTGGCCGGATTTGGGCGGTTGATGGCTTGTTTTGAGCCGGCGTCCATCAGCTGCCTCCGTTTTTGGCTTCCTCTTTGCCGGATCTGAAGACTGATTTAGCTTTTGGAGTGGTTCAAGGAAGAACGTCGAGGGAAGTAAGGGTTAGTTTTGATGTCTCTCTTCTTGCTAGGTCGGGGAGAAGAGGTCCTGGGTCTCGGTTGTGGCGGATCTTCCTTTCCCGAAAGGAAAGTTCTGGGTTCGGTTTGTGTCTCGGCGACAGGTTGCGACCGGATTTGCTAGCTCCGGTCAAAGTCTTGCTGTTGAGGTCTTGTCCGCAAAGGAACCGTCCTTGTTAGCATCGGATCTGGAAGGCGAGATTCGTGTTTTCCGGTGTACTGCCTTCTTCAGATCTCACTTTGCTTGTGTTTCTGATTGGGCCATTTCTTTTTGTAACCCGTTAATTTTCTAGGCCCATCAGCCTAAGCCCTTTTTGGGAATTATTAATATATTAAAAAAAAAAAAAAAAAAAAAAAAAAAAAAAAAAAAACCAATAATGAATATA

>AlSadhu1-4 Araly1 scaffold_3: 1662010-1662618 strand=-

AAAACTTATTTGAAAAGAGAAATGCTAAAAAAAGCCTTTGAGAGGCCCATTAGAAATCGCTCTCTCCTCTCTCTTGCAACCCTAGCCGCTTTTTAATTTCTGAGGGAGAGGCGCACCTTTCCGGCAGTCTCGTCGGCGCGTGTGTGACCTTTCCCTCCCAATTTCAGTTTCTGTTCTTCAACGCCGGCTGATGGGTCAGAGGGTGAAGCGGTGGTGAGCGACTTTCTTCTCCGGTGGTGTGAGGTGTGGTTTGGTCCTTCATTCGATGCTTACGGTGGCTCTCCTTCACGGCAGCTGAGAAACGGTCCATGCGTTCTACTGGTTAAAGGACTCCGGTGGATTGGATCTGGTTTCTCACGGCTCGGCGGCTGATGAGTTATAGCTCCGGCGGCGGCGGAAGTTGAGTGATGGATCGGTGGGTTCGCCACTTTGCGAAGAGGTTAACAGTGGATCCTTGGCCGGATCCTTTGGCTAGTTTCGGATGATCCTTGGCCGGATCTTGGTGGTTGATGGCTTGTTTTGAGCCGGCGTCCTTCATCTGCTACCGTTTTTGGCTTCCTCTTTGCCGGATCTGAAGACTGGATCAGCTTCTGGAGTGGTTCAAGGGTGAACGTCGAGGGAGGTAAGGGTTAGTTTTGATGTCTCTCTTCTTACTAGGTCGGGGAGAAGAGGTCCTGGGTCTCGGTTGTGGCGGTTCTACCTTTCCCGAAAGGAAAGTTCTAGGTTCGGTTTGTTTTCGGCGACAGGTTGCGACGGGATTTGCTAGCTCCGGTCAATGTCTCGATGTCGAGGACTTGTCCGCAAAGGAACCGTTCTTGTTAGCATCGGAGATGGAAGGCGAGGATCGTGTTTTCCGGTGTACTGCCTTCTTCAGATCTTACTTCGCTGTGTTTTTCCGATTTGGGCCGTTCCTTTTTTGTAACCCGTTTATTTCTCTTGGCCCATCAGCCAAAGCCCAATTTTGGGATTTAATAATATATATATAAAAAAAAAAAAAAAAAAAAAAAAAAAAAAAAAAAAAAAAAAACTTATTTGAA

>AlSadhu1-5 Araly1 scaffold_1: 24954753-24955365 strand=+
AAAAAAAAAAAAAAAAAAAAAAAAAAAAAAAAAGGGA*CAATCGTTCC*CATTGAAACCCTCTCTTCTCTCTCCTAAAGCCAGATCTTAGATCTACTTCATCTCCTTTTTTCTTTTTTCGTTTATTGATCTTCAGTGTCGGTTGATGGGTGAGGGAGTGAAGCGGTGGTGAGCGTCTTTCTTCTCCGGTGGTGTGAGGTGTGGTTTGGTCTTTGGCTCATTACTTGTTGGTGGCTCTCCTTCACGGCGGCCAGAGAAGCGGTTCCTTCGTTCTGCTGGTTAAGGACAACAGTAGATTGGATCTTGTTTCTCACGGCTCGGCGGCTTGTGATTGTGGCTCCGGCGAGTAGCGGAAGTTGAGCTTTCGATCGGTGGGTTTCACCACTTTGCGAAGAGGTTAACGGTGGCTCCTTGGCAGGATCCTAGATGGCTAGTTTCGGGTGATCCTTGGCCGGATTTGGGCGGTTGATGGCTTGTTTTGAGCCGGCGTCCATCAGCTGCCTCCGTTTTTGGCTTCCTCTTTGCCGGATCTGAAGACTGATTTAGCTTTTGGAGTGGTTCAAGGAAGAACGTCGAGGGAAGTAAGGGTTAGTTTTAATGTCTCTCTTCTTGCTAGGTCGGGGAGAAGAGGTCCTGGGTCTCGGTTGTGGCGGATCTTCCTTTCCCGAAAGGAAAGTTCTGGGTTCGGTTTGTGTCTCGGCGACAGGTTGCGACCGGATTTGCTAGCTCCGGTCAAAGTCTTGCTGTTGAGGTCTTGTCCGCAAAGGAACCGTCCTTGTTAGCATCGGATCTGGAAGGCGAGATTCGTGTTTTCCGGTGTACTGCCTTCTTCAGATCTCACTTTGCTTGTGTTTCTGATTGGGCCATTTCTTTTTGTAACCCGTTAATTTTCTAGGCCCATCAGCCTAAGCCCTTTTTGGGAATTATTAATATATTAAAAAAAAAAAAAAAAAAAAAAAACTAGGGAACACCGGTTTAGCCTGTTAATGGATTAATG

>AlSadhu1-6 Araly1 scaffold_4:841417-842023 strand=+ AAGATATACATTAGTTTCAGAGGCCCATTAGAAATCGCTCTCCCCTCTCTCTTGCAATCCTAGCCGCTTTTTAATTTCTGAGGGAGAGGCGCACCTTTCCGGCAGTCTCGTCGGCGCGTGTGTGACCTTTCCCTCCCAATTTCAGTTTCTGTTCTTCAACGCCGGCTGATGGGTCAGAGGGTGAAGCGGTGGTGAGCGACTTTCTTCTCCGGTGGTGTGAGGTGTGGTTTGGTCCTTCATTCGATGCTTACGGTGGCTCTCCTTCACGGCAGCTGAGAAACGGTCCATGCGTTCTACTGGTTAAAGGACTCCGGTGGATTGGATCTGGTTTCTCACGGCTCGGCGGCTGATGAGTTGTAGCTCCGACAGGCGGAAGTTGAGTGATGGATCGGTGGGTTCGCCACTTTGCGAAGAGGTTAACAGTGGATCCTTGGCCGGATCCTTTGGCTAGTTTCGGATGATCCTTGGCCGGATCTTGGTGGTTGATGGCTTGTTTTGAGCCGGCGTCCTTCATCTGCTACCGTTTTTGGCTTCCTCTTTGCCGGATCTGAAGACTGGATCAACTTCTGGAGTGGTTCAAGGGTGAACGTCGAGGGAGGTAAGGGTTAGTTTTGATGTCTCTCTTCTTGCTAGGTCGGGGAGAAGAGGTCTTGGGTCTCGGTTGTGGCGGTTCTACCTTTCCCGAAAGGAAAGTTCTAGGTTCGGTTTGTTTCTCGGCGACAGGTTGCGACGGGATTTGCTAGCTCCGGTCAATGTCTCGATGTCGAGGACTTGTCCGCAAAGGAACCGTTCTTGTTAGCATCGGATATGGAAGGCGAGGATCGTGTTTTCCGGTGTACTGCCTTCTTCAGATCTTACTTCGCTGTGTTTTTCCGATTTGGGCCGTTCCTTTTTTGTAACCCGTTTATTTCTCTTGGCCCATCAGCCAAAGCCCAATTTTGGGATTTAATAATATATATTTAAAAAAAAAAAAAAAAAAAAAAGATATACATTAGTT

>AlSadhu1-7 Araly1 scaffold_3:13675822-13676434 strand=+
AAGTTTCTCCACTAAAAAAAGGGA*CAATCGTTCC*CATTGAAACCCTCTCTTCTCTCTCCTAAAGCCAGATCTTAGATCTACTTCATCTCCTTTTTTCTTTTTTCGTTTATTGATCTTCAGTGTCGGTTGATGGGTGAGGGAGTGAAGCGGTGGTGAGCGTCTTTCTTCTCCGGTGGTGTGAGGTGTGGTTTGGTCTTTGGCTCATTGCTTGTTGGTGGCTCTCCTTCACGGCGGCCAGAGAAGCGGTTCCTTCGTTCTGCTGGTTAAGGACAACAGTAGATTGGATCTTGTTTCTCACGGCTCGGCGGCTTGTGATTGTGGCTCCGGCGAGTAGCGGAAGTTGAGCTTTCGATCGGTGGGTTTCACCACTTTGCGAAGAGGTTAACGGTGGCTCCTTGGCAGGATCCTAGATGGCTAGTTTCGGGTGATCCTTGGCCGGATTTGGGCGGTTGATGGCTTGTTTTGAGCCGGCGTCCATCAGCTGCCTCCGTTTTTGGCTTCCTCTTTGCCGGATCTGAAGACTGATTTAGCTTTTGGAGTGGTTCAAGGAAGAACGTCGAGGGAAGTAAGGGTTAGTTTTGATGTCTCTCTTCTTGCTAGGTCGGGGAGAAGAGGTCCTGGGTCTCGGTTGTGGCGGATCTTCCTTTCCCGAAAGGAAAGTTCTGGGTTCGGTTTGTGTCTCGGCGACAGGTTGCGACCGGATTTGCTAGCTCCGGTCAAAGTCTTGCTGTTGAGGTCTTGTCCGCAAAGGAACCGTCCTTGTTAGCATCGGATCTGGAAGGCGAGATTCGTGTTTTCCGGTGTACTGCCTTCTTCAGATCTCACTTTGCTTGTGTTTCTGATTGGGCCATTTCTTTTTGTAACCCGTTAATTTTCTAGGCCCATCAGCCTAAGCCCTTTTTGGGAATTATTAATATATTAAAAAAAAAAAAAAAAAAAAAAAAAGTTTCTCCACTAAA

>AlSadhu8-1 Araly1 scaffold_1:13276396-13277158 strand=-

AAATACTATGGTAGTAGTTCTTTAACAGAAAAAAAAAAAAAAAATGAGAGCA*CAATCGTTGC*ATCGATAATTGTGCCATCTCTCTCTCTTGAAGGCGATTTCCGGTTTCAGATCTAGGTTTTTTCAGTTCTTTTTCTGGTCTTAACGGCGGCGGAGGCCTCTCGAAGGTGGCCCAGGAGTGAGTCTTTGTGCTTCTCCGTCAACAGAGGTGTGGTGCTTCCCTGTTGATGTGGTCGTGGCTCTGGAATGGCGGAGGAGGAGCATTGATGCAGTGATCTCCTGGTTGAGGACGCTGGGAGTTGTATCGATGCTCCTCAGGCTCGACCGTCGGACTGGAAGCTGCGTCCACGGCGGAAGTTGGCGGGGATAAGGTGGGGCTGTTGTTTTGACAGGTGGCTAGAGGTGGATCCTTAGGATCTACGATGGCTGATGTGGGGGTCGGATTTAGAGGTTCTGGGCGAGCCACCGTCCTTTAGATCTGCCGCATCGGGTGTGCCGACTGTCCGGATTGAGGCATGAAGACTCTGGGACTTCGGAGCTCTCTGGCGAAGGAAGAGAGAGGATAACTCCAAGCGTGACGGGTTGCTAGGTCGGGATCCGTTACCGTCGACCCTGTCGTCGGAGTGACTAACTTTTTGGGAGGCTTGTGGTGGGTTGGTGCGACGGAAGAGCGGGATGGCTGTCGGAAACTAGAATCTTCCTCCAATTTTCTCAATCCTCTGACGCGGCTACGAAGCTGTGGACCTTGGTTCTACCAGATCTAAGGTTTGGTCACTCTGCGAACGATGATCAACAGGAGTCGATTCTTTTTGCTTTTTCCGGCGAGTTTCTGGTGTACCGGTTTTGTTACTGGGTTTTAATTCGTTGTAAGCCGAGGCCCATGCCTATTTGGGTTGATTTTATATAAAATTATTAATTAAAAAAAAAAAAAAAAAATACTATGGTA

>AlSadhu8-2 Araly1 scaffold_3:807549-808169 strand=+
TCAACGATTGAAAAACA*CAATCATTGC*ATCGATAATCGTGCCATTCTCTCTCTCTTGAAGGCGAATTCCGAGCTTTCGATCTAGTTTCTCCGGTTCTCTGTTCTTATCATCGGCTGAGATCCTTCGAAAGTGGTCTAGGTGTGAGTCGTGGTGGTTCTCCGTCAACAGAGGTGTGGTGTTTCCCCGCTGATGTGGTCGTGGCTCTGGAATGGCGGAGGAGGAGCTTTGAAGCGGTGGTCTCCCGGTTGAAAATGCTGGGAGTTGCTTGGATGCTCCTCAGGCTCAGCCGCCGGCCTAGTCGCTGCCTCTACGGGGAAGTTGGTGGGGATAAGGTGGTGTTTTTGTTGTTTTGACAGGTGGCTAGCGGTGGATCTAGGATCTACGATGGCTGATGCGGGGGTCGGATTTAAAGGAATTGGGTGAGCCACCGTCCCTTAGATCTGCCGTATTGGGTGCGCCGACTGTCCGGATCGAACCATGACGACTCTGGAATTTCGGATTTTTTTCGGTGAAGGAAGAGAGAGAATAACTCCAAGCGTGATGGGTTGCTAGGTCGGGATCCGTCACCGTCGAACCGCTTCGTCGGAGAGACTAACTTTATGGGAGGCTTGTGGTGGGTTGGTGCTTCGGAGGAGAGAGATAGCTGCCGGAAACCGAAATCCTGCATCGGTTATCTCAATCTCCCGATGCGGCCACGAAGCTGTCAGCTTTGGTTTCGCCGGATCTAAAGTCTGGTTTCTCTGCGAGCGATGATCAACAGGGGTCGAGTCGATTTTCTTTTTCCGGCGAGTTTTCCGGTATACCGTTTTGTAACCGGGCTTTCTTTTGTTGTAGGTTAAGCCTTGGCCCATTAGGGTTGAATTTTTAATATATAAAAAAAAGTTTATGATAAATAAATCGA

>AlSadhu8-3 Araly1 scaffold_2:25942-26642 strand=+
TATCCTTGTGAAAGGCA*CAATCGTTGCC*TCGATACACGAGCTATTCTCTCTCGTGAAGGCGATTTTCTAGATCTAGATCTAGTTTCTCCGGTGAATCTGGTTTAATCGAAGGTGAAGGTTTCTCGAAGGTGGTCCAGGAGTGAGTCGTCTTATCTCTCCGACAACAGTGGCGTGGTGTCTCCTGCTGATGTGGATGTGGCTCTGGAAGGGCGGAGGAGGAGTGTTGATGCAGTTGCCTCTCGGTTAGGGACGCTGGGAGTTGTGTCGGTGCTCCTCAGGCTCAACCGCCGGCCCATATGCTGCGTCCACGGCGGAAGTTGGCGAGAGGTAAAGTGGGTTTTGTGTTGTGTTGACAGGTGGCTAGCGGAGGATCTTTGATCTTTGATGGCTGATGCGGGGGTCGGATTTAAAGGTACAGGGTGAGCCACCGTCCTTTAGATCCGCAGGATCGGGTGTGCCGACTGTCCGGATCGAGGCTTGACATCTCAGGGATTCTGGATTTTCACGGAGATGGAAGAGCGAGGATATCCCCAAGCGTGACGGGTTGCTAGGTCGGGATTCGTCGCCGTCGAACCGTCTCGTCGGAGACACTAACTTTCTGGGAGGTTTGTGGCGGGTTGGTTCATCGGAGGAAAAGGAGGGCTGTCGGAAACCGAAATCTTCCACCGGCAATCTTATTCCTCTGATGCGGCCACGAAGTTGTGAGCCTTGATTTCGCCAGATCTAAGGTTTGGTGTCTCTGCGAACGATGATCAACAGGGGCCGAGTCGTTCTGGTTTTTCCGGCGAATTTCCGACGTTCCTATTTTGTAACTGGATTTCTTTTTTTCGTTGTAGGCCTTGGTCCATGCCCATTTAGGAAGAAAATTTTAATAAAATTATTTAAAAAATAATAATAATAATAAATAAAATAAAATTATCCTTG

>AlSadhu8-4 Araly1 scaffold_8:14602238-14602861 strand=+
AAAGTTACTTTTTACGTTCTTCGAAAAAGCA*CAATCGTTGC*TTCGATATACGAGTTTTCTCTCTCTCTTGAAAGCGATTTCTGAGATCTCGATCAAGTTTTTCCGGTGAAGCTGGTTTAATCGAAGGTGGTCCAGGAGTGGGTCGTCTTGTCTCTCCGACAACAGTGGTGTGGTGTCTCATGCTGATGTGGATGTGGCTATAGAAGGGTGGAGGAGGAGTGTTGATGCAGTTGCCTCTCGGTTAAGGACGCTGGGAGTTGTGTCGGTGTTCCTCAGGCTCAACCGCCGGTCCATAGGCTGCGTCCACGGTGGAAGTTGGTGGGGGTAAGGTGGATTTGTGTTATGTTGACAGGTGGCTAGCGGAGGGTCTTTGATCCTTGATGGCTGATGCGAGGGTCGGATTTAAAGGTACTGGGTGAGCCACCATCCTTTAGATCCGCCGGACCAGGTGTACCGACTGTCCGAATCGAGACTTGACGACCTAGGGATTCCGGATTTTCACGGAGATGGAAGAGCGAGGATAGCCCCAAGCGCGACGGGTTGCTAGGTCGGGATTCGTAGCCGTCGAACCGTCTCGTCGGAGACACTAACTTTCTAGGAGGTTTGTGGCGGGTTGGTTCATCGGAGGAAAAGGAGGGCTGTCGGAAACCGAAATCTTCCACCGACAATCTTGTTCCTCTGATGAGGCCACGAAGTTGTGAGCCTTGATTTCGCTAGATCTAAGGTTTGGTGTCTCTGCGAACGGTGATCAACAGGGGCCGAGTCGTTCTGGTTTTTCCGGCGAGTTTCCGGCGGTTTCATATTTTGTAACTGGGCTTTTCTTCGTTGTAAGGCCTTGGCCATTGCCCATTTGGGAAGAGTTTTAATAAAGTTATTATTATAAAAAAAAAAAAGTTACTTTTTAC

>AlSadhu6-1 Araly1 scaffold_3:21898960-21899646 strand=-

AATATAGTCAGTGTGTCGGCA*CAATCGTTGC*CTTGAAATCCGCGCTTTTCTCTCGCCTTGAAGACGATTTTCTTTTCCTAGATCTAGTTCGCAGATCCTTGTAGCGCTTCAATAACCAGCCGAATCTACTCTTGGACGATTCAGGAGAGAGTCGAGGCGTATCTCCGTCAACGGTGGTGTGGCGTTTTCCCTTCGATGTGGAGGTGACTCGGGTCTGATGGAGGAGGGCTACTGCAGCGGATCTCTCATGGTTGGGACGTCGTGGGTGGCTTCAGTTCCTCCGGCTCAACCGTCAGTCTCAAGTTGCGTTCACGGCGGAAGTTGGGGGGATTAAGGTGGGTTCGTTGTTTGCTCGGTGGTTAGCTGCAGATCCGTAGGATCTTAGAGGGCGGTTGTGGATGTCGGGTGTAAGGGTTTTGGATTTGAGCCACCATCCTTTAGATCCGCCACAACTTAGCATCCATTCGAGCCGGATTGATTCGTCAAAGCTCAGGGATCTTGGTTTGTTTTTCCGGTCTGGAAAAGAGGGGTTTCCGTCCAGTGTGTCGGATTGCTTCTTCAGGATCTGATGCCGTCGATGTGCTTAGTCGGAGTGACTAGCTTTCCGGGAGACTTGTGGTGGGCTGGTTCGTCGGAGGCATGTAATGGATGGCGGAAACCGGTCTCTTTCGCCGAAATTTCACGGTCTTCGGTTGTAGCCACAAAACTGTGGGTCTTGAGCCCGCCGGATTTCAGGTTCCGGTTGCTCTGCGGGCATCTATCAACAAACGGCGAGCCTCTTTTGTTTTTCCGGTGAATTGCCAATTTCCTTTTTGTAACTGGATTGGACTTTTGTAGATTCGGCCCAAGCCCATTTGGGAGAAAAAAAAATATATATATAAGTAGTTATTTAAAAAAAAAAAAAAAAAAAAAAAATATAGTCAGTGT

>AlSadhu3-1 Araly1 scaffold_7:12620425-12621361 strand=+
AAAAAAAAGAAGCAAATGGAGGA*CAATCGTTCG*GCGTCTCTCTTCTCTCTCTTCAAGAAAAAAAAAAGCAGATCTTCTTTAAGTTTTTCTTTTTTTTTGCTTTTAGATCTTCATTGCAGGTCTGGTTCTTCTTCAATGATGCGGTGGTGAGCGAGCTTTCCTCTCCGACAGCTGCGGTGTGGAGTATCCGGTTCGTGATGCATTTGTGGCTTCTTCCTCGGCAGTTTTGAGAAATCTCCCGATGCTAAATCTTTTGGTTGAGGACGCTGGAGGCATGGTGGTGGAGATTTCTCGCGGCTCGTCTGCTCGCGGTGGGTGCTGCGGGTTGCGGCGGAAGTCATGAAGGCGGATTGGTGGGTTAGGCTGTTTATTGAGGCAATTAACGACAGATCCATCTGGATCTGATACGGCTAATCTCGGATCTTGGCTGTTGAGGCTTGGTTTGAGCCATTTAACCTCTCAGCTTCCTAGATTTTGTTTTCGTCTGACCGGATCTGAGGTTGGCTTTGCTCGTGAAGGTCATGTTTGCTTGTCAGCGCTGGAGATTGGGTGTTAGATTTTTAGGTTCTCTCTTTTCGCTAGGTAGAGGATTAGAGAGATTGGGTTACGGATCTTCTGGGAATCGGTTCATGCCGGGAGAAGTGTTGCAGGTTCATGTTTGTCGCCGGGATCGGCTTTGCGACGGGAACCGATTACTTCTGCCAATGAGTTCGTTTTCGCCGTCGTAGCCGCGAAGCAGTGCTTCTCGTAATCGCTGGAATTAATGGATGTGTTTCCTTGTTTTCCGATCAACCTCCGGGTCCGATCTAGTGTCACCGGAATTGTCAGCCGTTTCTTTCTTTTTTTGTATCCGTGTTATCGTTGTAAGGCCTTGCTTATTGCCCTTAGGCCTAAAGCCCGTTTGGGATGAAGGTATTAACATA
AGCATTTAACGTCGAAAAAAAAAAAAAAAAAGAAGCAAATG

>AlSadhu10-1 Araly1 scaffold_3:4473616-4474232 strand=+
AAGACTAGACTCTAGTATGTGAAAAGAATCGCA*CAATCGTTGC*CTCGGGAACTGAGCTTCTCTCTCTCTTGCAGAAGTTTTTTCAGATTTAGATCTAGTTTTTCCGATATCAGATCTTCATCCCTGGCCAAGGTCCTTTCACAGTGGTCCAGGAGTGAGCTTGCTTTTCTTCGCCGTCAGCGGTGGAGAGGATAATCCCCATCGATGTGGTCGTCGCTCCGGTTTGGCGGTGGAGGAGCTCCGCAGCGTTGGTTCCACGGTTTGAGGACGCCGAGGAATGCTATGTAGATCCTCTTGCTCAGCCGTTGATATGGTGGTTGCGGCTACGGCGGAAGTTGGTGGTGATTCGGTGGGTTTCGTTTGTTTGGCGAGATGGTTAACTGTAGATCTTGGCAGATCTTAGATGGCTAATGCGGAGGCAAGGGTTCAGAGTCTTTGGCTGAGCACCAATTTCTGGATCCGCTGTGTTTGGCTTCCATCCGCCCGGTTGAAGGATAGCAGCTCAGGGAGATCAGAGAAATCACGGAGAGAGAAGAAAGGGGATAAACCCCAGTGTATCGGGTTGCTAGGCATGGAACAGATACCGTGGAACCGGTGAGTCGGAGTGGCTAGCTTTCTGGGAGAGTTGTAGTGGGTTGGTGCGGCGGCGGAGAGAGTTCGCAAACGGAAACCGGAATCTTCCGACTGCGTTTCTCGATCTTTCGTGTGCGTCCACAAAGCCGTGACTTTTGAGTCCGCCGGATTTAAGGCTCCGGCCACTCTGCTTCCGATCGTCAACAAGGTTCGAGCTTTTTTTAACTTTCCGACGATTTTCTCCGATTAACCTTTTTGTAACTGGATTTCTTCTGTGTTTGGGCCTTAGCCCATGCCCATTAATGGGTTGATTAATAAAATTATATTAAAAAAAAAAAAAAAAAAAAAAAAAAAAAAAAAAAAAGACTAGACTCTAGTA

>AlSadhu10-2 Araly1 scaffold_2:9675105-9675721 strand=+
ATGGGCAAAAAAGTAACACAGTGTCATAATGTGAAAAGAATCGCA*CAATCGTTGC*CTCGGGAACTGAGCTTCTCTCTCTCTTGCAGAAGTTTTTTCAGATTTAGATCTAGTTTTTCCGATATCAGATCTTCATCCCTGGCCAAGGTCCTTTCACAGTGGTCCAGGAGTGAGCTTGCTTTTCTTCGCCGTCAGCGGTGGAGAGGATAATCCCCATCGATGTGGTCGTCGCTCCGGTTTGGCGGTGGAGGAGCTCCGTAGCGTTGGTTCCACGGTTTGAGGACGCCGAGGAATGCTATGTAGATCCTCTTGCTCAGCCGTTGATATGGTGGTTGCGGCTACGGCGGAAGTTGGTGGTGATTCGGTGGGTTTCGTTTGTTTGGCGAGATGGTTAACTGTAGATCTTGGCAGATCTTAGATGGCTAATGCGGAGGCAAGGGTTTAGAGTCTTTGGCTGAGCACCAATTTCTGGATCCGCTGTGTTTGGCTTCCATCCGCCCGGTTGAAGGATAGCAGCTCAGGGAGATCAGAGAAATCACGGAGAGAGAAGAAAGGGGATAAACCCCAGTGTATCGGGTTGCTAGGCATGGAACAGATACCGTGGAACCGGTGAGTCGGAGTGGCTAGCTTTCTGGGAGAGTTGTAGTGGGTTGGTGCGGCGGCGGAGAGAGTTCGCAGACGGAAACCGGAATCTTCCGACTGCGTTTCTCGATCTTTCGTGTGCGTCCACAAAGCCGTGACTTTTGAGTCCGCCGGATTTAAGGCTCCGGCCACTCTGCTTCCGATCGTCAACAAGGTTCGAGCTTTTTTTTTACTTTCCGGAGATTTTCTCCGATTAACCTTTTTGTAACTGGATTTCTTCTGTGTTTGGGCCTTAGCCCATGCCCATTAATGGGTTGATTAATAAAATTATATAAAAAAAAAAAAAAAAAAAAAGATGTCATTACAGAATCCTTTAACAAATTTAGCTGATGTTTACATTGCAATTTGTCTTACA

>AlSadhu5-1 Araly1 scaffold_7:17697122-17698008 strand=+
AAAATATGAAATTTAAAATGGA*CAATCGTTCC*CGAAAAACCTCTCTTCTCTCTCCTTACTGTAGCAAGTTTTTAGATCTAGATCTGCTTTTTTTTCCGGTGGCCTTTGATCTTCATCTCTTCGGTCTTACTTTGAAAAAACAGGGGTGAACAAGATCCCTTCTCCGTCGGCAGCGGTGAGGTGTTCTCCCCTTCGATGTGGTTACGGCTCCGCCGTGTCGGAAGTAGAGATTCGCTGCGGTCGCCTTCGGTTGAGGACACCGTTAACCTCCTGGCTCAGCCGTCGTCTCGGTCTCTGCAACTACGGTGGAAGTCGATGGGGAGAAGGTGGGTAGTGCTGAATCGCCATGGAGGTTAACGGTGGATCGTCGTGGATCTTGTTGGCCGTCGCGGATTCAATGTGTTTGGGTTTTCGATTGAGCCATCGGCCTCAAACACCACCGCGATCTGCTTCCTCCTGGCCGGATTGAAGGTTTTTGGTTCGGAGAATCAGAGAATCGCCGGAATTGGGAAGGAGGGGTTATGTCTGAGTTTGTCGGCTTGCTAGGTACGGGAGCTGACAACGGCGGGCCGTGACGTCGGAGGGTTGACCATACCGAGAGATCTGTGGCGGGAACGGTTGGTCGAGGGAAAAGGAACGGAATCGAGAGTCGGATGCTTCCTCTATGTTTACTAGATACTTCGATCCGTCGCAAAGCTATGGTTCTCGCATCCGCCGATGCTAATGGTCGGACTCTCTGCACCACGGTGTCACAAGGGCAGGATCCTCTTTTCAGACACCGACAATATTTTCCGATTTGCCTTTGTCTCTGTTTTTCACTGTAATCCGAGGCCCAACCCAATTTAGTGGGTAACCTATATTAATAAAAGGCCCGTTTCAAAAAAATAAAGAAAATATGAA

>AlSadhu8-5 Araly1 scaffold_7:17882685-17884241 strand=-

ACAACATGAACGTATTAAATTTATCTCATCGATTGAGATAACAAATTTTTATGTGCCATTCTCTCTCTCTTAAAGACGATTTCCGGTTTTCAGATCTAGTTTTTTCAGGTCATTTTCAGGTCTTAACGACGGCGGAGGTCTCTCGAAGGTGGCCCACGAGTGAGTCTTTGTGCTTCTCCGTCAACAGAGGTGTGGTGATTCTCCGTTGATGTGGTCGTGGCTCTGGAATGGCGGAGGAGGAGCATTGATGTAGTGGTCTCCCGGTTGAGGATGCTGGGAGTTGCTTCGATGCTCCAGAGGCTCGGTCGTCTGCCTAGAAGCTGCGTCTAC*TAGGCCTGGGCATAATATCCGAACTCGACCCGAAAAACCCGATCCGAATCCGAACCCGAAGTTTTAAAATACCCGACGGGTTCTAAACTTCAAAACCCGAAAACCCGAACCCGACCCAAACCCAAACCCGAACAAGTACCCGAGCATATGCAAAATATAAGTACATAGTTATAATATATTAGTAATATTTATACTTGTAATAACCCAAGTGCCTAAATTATTCAGATTTTAAGATATTTTTGGGTGTTGTAGCTATTTTGGGCTAGATTCGGGTATAAAATACTTGGGTTTTATAGATTTTTTGAGTATTTTTAATACGTTGCATAGAATCGGATACAAAATTTCTAATATTTGGATACTTCGGGTAAACCCGGTAAACCCGAATCCGAACCCGAAATACCCGATCCGAACCCGAAGTTTTAAAATACCCGAACGGGTCTTAGTTTTCTAAACCCGAAAAACCCAAACCCGAAAAACCCGACCCGAATCCGACCCGAATATCCAAACGCCCAGGCCTA*GCGTCTACGGCGGAAGTTAGCGGGTATAAGGTGGGTATGTTGTTTTGACAGGTGGCTAGCGGTGGATCCATAGAATCTTCGATGGCTGATGCGGGGTCGGATTTAGAGGTTCTGGATGAGCCACTGTCCTTTAGATCTGCCCTATCGATCTGCCGTATCGGGTGAGCCGACTGTCCGGATCGAGGCATGAAGACTCTGGGACTTCAAAGTTTTCCGGTGAAGGAAGAGAGAAGATAACTCCAAGCGTGACAGGTTGCTAAGTCGGGATCCGTTACCGTTGAACCGCTTCGTCGGAGTGACTAACTTTCTGGAAGGCTTGTGGTGGGTTGGTGCGTCAGAGGAAAGGGATGGCTGACAGAAACCAGAATCTTCCACCAGTTTTCTCAATCTTTTGACGCGGCCACGAAGCTGTGGGCCCTTGATTCTGCCAGATCTAAGGTTTCGGTCACTCTGCGGACGATGATCAACATGATCGAGTCTTTTTTCTTTTTTCGGCGAGATTCGGTGTACTTTTTTGTAATTGGGTTTCAAATTCTATGTAGGCCTATTTCTAGTCCAATTGGGTTGAATTTTATAAATAAACTATATTTAAAATATATATATATTTTATTATTTTGCCGTAAAAAAAAATAAAACAGATATATATTATTTACGGTTAA

>AlSadhu8-6 Araly1 scaffold_6:17227119-17227908 strand=-
TCCGGAGCA*CAATCGTTGC*ATCGATAATAGTGCCCTTTTCTCACTCTTGAAGGCGATTTCGAGTTTTAGATCTAGGTTTCCGGTAACTCTGGTTTAATCGCCGGCTGAGGTTCCTCGGAGGTGGCCAGGAGTGAGTCATGTTGCTTCTCTGTCAACAGAGGTGTGGTGCTTCCCCGCTGATGTGGTCGTGGCCCCGCTGATGTGGTCGTGGCTATGGAATGGCGGAGGAAGAGCTTTGAAGCGGTGCCTCCCGGTTGAGGACGCTGGGAGTGGCTTCGATGCTCTTCAGGCTTAGCCGTCGGCCTAGAAGTTGCGTCTACGGCGGAAGTTAGCGGGGATAAGGTGGGTTTTGTTGATTTGACAGGTGGCTAGCGGTGGATCTGAGATCTGCGATGGCCGATGCGGGGGTCGGATTTAAAAGTTCTGGGTGAGCCACCGTCTTCTAGATCCGCCGTATCGGGTGTGTCCACTGTCCGGATCGAAGCTCATGACTCTGGGATATCGGAATCTCCGGTGTTGGAAAAGCGAGGTTAACTCCAAGTGTGACATGTTGCTAGGTCAGGATCTGTCACCGTCGATCCGTGCCGTCGGAGGACTAACTTTCTGAGAGGCTTGTGGTGGATTGGTGCGTCGGAGGAGAGAGATAGCTGCCGGAAACCGAAATCTTCCTACGGTTATTTCAATCTCCCGATGCGGCCACAAAGCTGTCGGCCTTGATTTCGTCGGATTTAGGGTTTGGTCTCTCTGCGGTCGGTTATCAACAAGGGTCGAGTCGCTTTTCTTTTCCGGCATTTTTCTGGTGTACCGGTTCTGTAACTGGGCTTTATTTTCGAGTGTAGGCCAAGGCCCATTGCCCTTTTGGGTTGATTGAATAAAAAAAAAAAAAAAAAAAAAAAAAAAAAAAAAAAAAAAAGAGATCAGCC

>AlSadhu6-2 Araly1 scaffold_2:14183205-14186662 strand=+
AAACTGGCAAAATTCGGTTCGAAACTGAAGAATTATAAGAGAAGCA*CAATCGTTGC*CTTGAAATCCGCGCTTTTCTCTCGCCTTGAAGACGATTTTCTTTTCCTAGATCTAGATCGCAGATCCTTGTAGCGTTTCAATAACCAGCCGAATCTACTCTTGGACGATTCAGGAGAGAGTCGAGGCGTATCTCCGTCAACGGTGGTGTGGCGTTTTCCCTTCGATGTGG*ACACTACAAAAAAAGAAGTGGATTGTATCACTTAAATAGTATCAGAAAAATAAGTGATTTTAAATTAAATCATTTATTAATAAATGATAAAAAAAAAATATGTGGGTTAACATCAATTATTATAATTGATGTTAAAATTGAATTGTTTAACATCGCTTCAATTAAAATGATACAAATTTATATTACTTTGCATCACTTCAAAAATATTGATACAAAATTATGAATTTATATCAATTAAATACTTGATGTATTTATAAATTTTATTTATATCACTTACAATTGATACATATTTAATATCAATTAGAAAATGACACAAATTTATAGTAGTTTGCATCACATCAAAAAAATTGATACAAAGATATTAATTCATATCAGTTAAATAATTGATGTATTTATCATTTTTGTTCACATCACTTAAAAATGATACAAAATTAACATTAATTATATAAACGATAGAAAATTGATATCAATAATACACAGATTCGACAATTAGCATGTAGTTTTACTTTGTCTCCGTTGCAAATTTTGATGCTACTTAATCTTAATTTTTGATATTTAGATTAGTTAGTTTATTTAAAAACAAAAGAAGATTTGAAAGAATTAAGCATGTTGGGAATATTCACAACAAAGGTTTTTTTTTTAAATAGCAAGTTTAAAAAACGCTCAAATATTTTTAAAAATAACTAATCTATATCTCAAAAATCTTATCCATAAATAAACAGTTACATTTGGGTTTTATATTCACAAACGTAGGCTTTTAGTTAGTATCCATTTCTTTTTCTTTATTTTCGTAACAATTGTTTTTTTTTACTTTCAATTTTGTATTTAAACCTGTTTTGTGAAAAAAAAACTTTGTTTCCACTACTCAATCTACTCCTTAACCAACCCACGATTTCTGTAACTTCTTAAATAAAAATAATATCTTATGTGATTTTAAATATAATTAAAGAGCTTTTTCGTTTAGTATAAATCTTAATTTTGTCAAAATTCTTTTCCTAAATCGACAGAAACAACAAAAAAATGTTTGGAGTCACCAGAACATAATACTCTTTCTTCTCTAAGTCCACGTGAAATTTTGTTTGAACGATGCCAACAATGCACTTTTGGTGAATCAAGTTATGTCCAACACATAGTCATATTCCTCACTTAGTCTTCTAAATCATTCCAAGGCAAATATTATTTTTCTTCTATAATTTACATTGTTGATTTTCTTAGGTGTGATTCTTATTTTTCTCTGTTATTTTTGTTTTCATACTTAGAAAATCTGAAGATGAAGGATCAAATTAAGACAATGGAAAGGAAGGAGCAGTGATCAATATCCTCAAGACAATGGAAAAGAAAGAGCATGGAATGAAGAAAAATCTGTTTCATATACCACTTTAACATGAAGAACTCATAGCTTGGTCATGATCTAAGTTCATATAATTTTTTTTTTCTTTTGTAAAATAGCTAATGATCTTCACGAAGTACTAATGATCGAGTCTGCATTGATTGATTTGTATTCATGATTTTTTTTTCATTTTTCGTAAATGAAAATTTGTTTTTTTAATGATTATTAGTAAAAATTAAAATCTTATCAAGATTTTAGTAGATATTATTGAAAACGATTTCATTTACAAAAAATTAATAATTAAAAAAATAGTAATTATGGGAAATATTTAAGATTATTAATCTTTTAATAATTTGGAAATTATTTAAATTTTAAACCTTATCAACATCACTTAAATAAACCGATGTAAAGGTTAACATCATTTTTTTCAACTGAAACAAATCCTAACATCACTTAAATAAACTGATGTAAAAGTTAACATCAATTTTTGCAATTGATACATATTAACATCGATTACAATAATCGATGTAAATATTTTGGATATTAACATCGTTTAGTAATAAATCGATACAAATTATTTGCATCACCACTTTCAGAGTCACTTATTTAATGATGTAAATACTTCATTCATAACTGATGTAAATATATAAAATAAATGATGTAAAATGACCTTTTTTTGTAGTGGGAGGTGACACGGG*TCTGATGGAGGAGGGCTACTGCAGCGGATCTCTCATGGTTGGGACGTCGTGGGTGGCTTCAGTTCCTCCGGCTCAACCGTCAGTCTCAAGTTGCGTTCACGGCGGAAGTTGGGGGGATTAAGGTGGGTTCGTTGTTTGCTCGGTGGTTAGCTGCAGATCCGTAGGATCTTAGAGGGCGGTTGTGGATGTCGGGTGTAAGGGTTTTGGATTTGAGCCACCATCCTTTAGATCCGCCACAACTTAGTATCCATCGAGCCGGATTGATTCGTCAAAGCTCAGGGATCTTGGTTTGTTTTTCCGGTCTGGAAGAGAGGGGTTTCCGTCCAGTGTGTCGGATTGCTTCTTCAGGATCTGATGCCGTCGATGTGCTTAGTCGGAGTGACTAGCTTTCCGGGAGACTTGTGGTGGGCTGGTTCGTCGGAGGCATGTAATGGATGGCGGAAACCGGTCTCTTTCGCCGAAATTTCACGGTCTTCGGTTGTAGCCACAAAACTGTGGGTCTTGAGCCCGCCGGATTTCAGGTTCCGGTTGCTCTGCGGGCATCTATCAACAAACGGCGAACCTCTTTTGTTTTTCCGGTGAATTGCCGATTTCCTTTTTTGTAACTGGATTGGACTTTTGTAGATTCGGCCCAAGCCCATTTGGGAGAAATTTTTTAATAAAAGTTAAAAAAAAAAAACTGGCAAAATTC

>AlSadhu6-3 Araly1 scaffold_8:5795744-5796493 strand=+
ATGTTCTAACACAACAATACTGCATAACGATATAAGTGTTCGAGCTCTCAACTTAGTTCATACGGAAAAGGCA*CAATCGTTGC*CTCGTTAGTCGTGTATTTCTCTCTCGTGAAGACGATTTTCAGTTCTCAGATCTAGGTTTCTTTACTGTTTTGTTCTTCAATCTTCGGTCGAATCTTCTCAAAAGCGGTCCAGGAGTGAGCCAGGGCGTTTCTCTGTCAACGGTGGTGTGGCGTTTTCCCTTCGATGTGGAGGTGACTCGGGTCTGATTGGAGGAGGTCTACTGCAGCGATTCTCTCATGGTTGGGACGTCGTGAGTGGCTCAGTTCCTCCGGCTCAACCGTCAGTCTCAAGTTGCGTTCACGGCGGAAGTTGGGGGGATTAAGGTGGGTTCGTTGTTTGCTCGGTGTTTAGCTGCAGATCCATAGGATCTTAGAGGGCGGTTGTGGATTTCGGGTGTACGGGGTTTGGATTTGAGCCACCATCCTTTAGATCCGCCACAATTTAGCATCCATCGAGCCGGATTAATTCGTCAAAGCTCTGGGATCTTGGTTTGTTTTTCCGGTCTGGAAGAGAGGGGTTTCCGTCTAGTGAGTCGGATTGCTTCTTCAGGATCTGTTTCCGTCGATGTGCTTAGTCGGAGTGACTAGCTTTCCGGGAGACTTGGGGTGGGTTGGTTCGTCGGAGGTATGTAATTGATGGCGGAAACCGGTCTCTTTCGCCGATATTTCACGCTCTTCGGTTGTAGCCACAAAACTGTGGGTCTTGAGCCCGCCGGATGTCAGGCTCTGGTTGCTCTGCGGGCATCTATCAACAAACGGCGAGCTTTTTTTCTTCCGGTGAAACACCGATTTGCTATTTTGTAACTGGTCTAGGCTTTTGTTATTAGATGATTTATATAAATAAATAAACCACTATATAATTTAATACATTTAAAACTACAAAATAATTTTCATGTAACAATATTTGTTATATTTAAGTTTGAATATAGAAT

>AlSadhu7-2-1 Araly1 scaffold_7:4360460-4360769 strand=-
AAGTTTTAATTATCAAGAGATACGGCC*CAATCGTTGC*CTCGATAGCTGTGCATTTCTCTCTCTCTTGAAGACGATTTCCGGCAACCAGATCTAGGGTTCTTTCGTTCCGTTCTGCTCTTCAATCTTCGGTTCGAGGCTTCTCCAAGGCGGTCCAGGAGTGAGTCGGAGCGTATCTCCGTCAACGGCGGTGTGGTGTTTCCTCTCTGATGTGGTTGTGGCCCTGGTTCTGACGGATGAGGAGTTTTGCAGCGGTGGTCTCGCGGTTGAGGACGCCGGGAGATGCTGCAATGCGCCTCAGGCTCACCCGTCGATCTAGTTGCTGCGTCCACGGCGGAAGTTGGAGGGGATAAGGGGGGTTCGTTGTCCGACGCGGTGGTTAGCTGTGGATCTGTTTGGATCTTAGAGGGCTGATGCGGGGTTCGGGTTTAGAGGTTCACGGCGAGCCAACGTTCTCTAGATCCGCCGCTTCTGGTCTCCACCGGTCCGGATCGATGCGTGAAGATTCAGGTTTTTTGGTGTTACCGGTTTTGGGAGAAAAGGGTTACCTCCCAGTGGGTCGGGTTGCTAGGTCGGGATCCGACTCCGTCGATGCGCTGCGTTGGAGTGACTAGCTTTCTGGGAGGCTTGTGGCGGGTTGGTGCGTCGGAGGAGAGACTCTTCTGACGGAAACCAGATTCTTCCGTCGGTGGTAGTCGATTTTCTGACGCGGCCACGAAGCGGTGGGTCTTGAATCTGCCAGATTTAAGGCTCCGGTTGCTCTACGAGCGTCTATCAACAAGGGGCGAGCTTTTCTTCTTTTTCCGGCGAGATGCCGATTTACCTTTTTGTAACTGGATTGGTTTGTGTGTAGGCTTCGGCCCAAGCCCGTTAGGGTGGTTTTAATAAAATATTTAAAAAAAAAAAAAAAAAAAAAAAAAAAGTTTTAATTATCAA

>AlSadhu5d Araly1 scaffold_6:5062639-5062768 strand=-
TCTGCCTCAAGTGCATCAGGGGTGAGTAAGATTCCATCTCCGTCGGATGTGGTGTGGAGTTCTCTCGTTCGATGTTGTTGTGGCTTCGATGTGGCGGTAGAGGTGAGCTGTTGCAGATCCTGCCTGGTTGAGGACGCCGGTATGGGTAGCGGTTCATCCTCGTTGCTCAGTCGTCGCACCGGCCGCTGCGGCAACTGCGGACGTCGTGGGGGAGACGGAGGGTTTTTGCCGATTTGTTGTGGAGGTTAACTGTGGATCTTTCGGGACCTAGATGGCTGGCGTGGAGAACGGTGATCTCTGCTGGATTGAGCCTCCGGTGTTTGCTACCGCTGCGTTTTGCTTCCTACTGGCCGGATTGATGGGATCTTGCTCTGTGAATCGGGATTATGTCGGGATTGGGAAGGAGGGGTTATGGCTGTGCTTGTCAGCTTGCTAGGTAGGGGAGCTGTCAACGGCGGGCCTGGTTGTCGGAGAGTTGACCTTACCGGAAGATTTGTGGCGGGTCTGGTGGGTCGTCGGAAAAGGAGCGCTGCCGGGATTCAGAAGCTTCCGTCATCGTCTCTAGATCCGTCGATTCCGCCGCAAAGCTGTGGATCTTGCTTCTGTCGGTGTTACTGCGTCGGATTCTCTGTTTCCCGGTAACCACTAGGTCACGAGCTTCTTTTCCAATTCCGATTAGGGTCTCCGATTTGCATTTTGTAACTGATGTATACTTGTTGTAGTTTCAAAGCCAAGCCCAATTGGGAAGCCCAATTTAATGAAAAGCCTTTTATTTAAAAAAAAAAAAAAAAAAAAAAAAATTACA

>AlSadhu5d2 Araly1 scaffold_6:25041036-25041746 strand=+
CACGTAAGTAGATCTAGGTTTTTTAGTTTTCTTCAGTCCTTCACCGCCGGTCTCGAGCCTGCCTCCGGTGCTCAGGGGTGAGAGCGTTCCTTTCTCCGTCGGCGGTGGTGTGGTTCTCTCCGGATCGATGTTTGTTGTGGCTCCGGTGTGCCGGAGAGGAGATCTGCTGCGGATCCTGTCTGGTTGAGGACGCCGGTATGGGCAGCGGATCTTCTCGTCGCTCACCCGTTGCGCCGGCCGCTGCGACAGCGGCGGAAGTCGTCAAGGAGAAGGTGGGTTAGTCGTTTGCCGTGGAGGTTAACCGTGGATCTTTGGGTCCTAGTTGGCTGGCGTGGAGTACGGTGTCTTTCTTCGGGATTGAGCCTCCGGAGTTTGCTGCCGCCGCGTTTTTGCTTCCTCCTTGGCCGGATTGATAGGTTCGCTTCTCTGTGAGTCGGGAAGTCGTCGGGATCGGGAAAGAAGGGTTATGGCTGAGTGTGGCGGCTAGCTAGGAAGGGGAGCTGTCATCGGCGGCTCGTGTTGTCGGGGAGTTGACCTTGCTGGGAGGTTTGTGGCGGGTTGGTAGGTCGTCGGAGAAGGCACGTCGCCGGGAATCAGAAGCTCCCGTCAACGTTCTGGATCCGTCGATTCCGCCGCAATGTTGTGGACCTTGCTTCTGCCGGCGTTTCTGTGTCGGACTCCCTGTTTCACGTTGTCCACCAGGCCAAGAGCTTCTTTTCCGGCGTCGATTTAGGGTTTCCGTTTTGCATTTTGTACCTGTGTTGTTGGATGTATGTTCTCGGGCCAAAGCCCATTTTGGGAAGGGGCCCTTTAATGAAAAGCCCGTTTAAAAAAAAAAAAAAAAACAACTAAGAGATGCATGGTTCGAGGCACGACCACTGG

>AlSadhu5d3 Araly1 scaffold_5:4156620-4157046 strand=-
TAATGAATATTGGGAGAGTCTTTGATATAATTAATTAATAAATGCTTGTAGGCTTGCTAGGTAGGGGAGCTGTCAACGGCGGGTCGGGTTGTCGAAGAGTTGACCTTACCGGGAGATTTGTGGCGGGTATGGTGGGTCATCGGAAAAGTAACGAGGCCGGGAATCAGAAGCTTCCGTCATCGTCTCTAGATCCGTCGATTCTGCCGTAAAGCTGTGGATCTTGCTTCTGCCGGTGTTACTGCGTCGGATTCTGCGTTTCCCGGTAACCACTAGGTCACGAGCTTCTTTTCCGGCTCCGATTACGTTTCTCGATTTGTTTTTTTTGTACCTGATGTTTCTTGCTATATTTTCAAGGCCAAACCCAATTGGGAAGCTCAATATCAATGAAAAGCCTTTAAAAAAAAAAAAAAAAAAGTTTGTAGCTTTAGCAGAATTA

>AlSadhu1d Araly1 scaffold_2:14298861-14299465 strand=+
TTTTTTTCTTTTTTTTTTTTTAATTTTAACTCTCAAATCTGAAATAAGAAGAAGCGGTGGTGAGCGACTTTCTTCTCCGGTGGTGTGAGGTGTGGTTTGGTCCTTCATTCGATGCTTACGGTGGCTCTCCTTCACGGCAGCTGAGAAACGGTCCATGCGTTCTACTGGTTAAAGGACTCCGGTGGATTGGATCTGGTTTCTCACGGCTCGGCGGCTGATGAGTTGTAGCTCCGGCGGCGGCGGAAGTTGAGTGATGGATCGGTGGGTTCGCCACTTTGCGAAGAGGTTAACAGTGGATCCTTGGCCGGATCCTTTGGCTAGTTTCGGATGATCCTTGGCCGGATCTTGGTGGTTGATGGCTTGTTTTGAGCCGGCGTCCTTCATCTGCCACCGTTTTTGGCTTCCTCTTTGCCGGATCTGAAGACTGGATCAGCTTCTGGAGTGGTTCAAGGGTGAACGTCGAGGGAGGTAAGGGTTAGTTTTGATGTCTCTCTTCTTGCTAGGTCGGGGAGAAGAGGTCGTCTCGGTTGTGGCGGTTCTACCTTTTCCGAAAGGAAAGTTCTAGGTTCGGTTTGTTTCTCGGCGACAGGTTGCGACGGGATTTGCTAGCTCCGGTCAATGTCTCGATGTCGAGGACTTGTCCGCAAAGGAACCGTTCTTGTTAGCATCGGACTTGGAAGGCGAGGATCGTGTTTTCCGGTGTTCTGCCTTCTTCAGATCTTACTTCGCTGTGTTTTTCCGATTTGGACCGTTCCTTTTTTGTAACCCGTTTATTTCTCTTGGCCCATCAGCCAAAGCCCAATTTTGGGATTTAATAAAATATATTAAAAAAAAAAAAAAAAAAAAAAAAAAAAAAAAAAAAAAGAAGAAGACTATATACAATTCTTTTTTTTCCATAAAAAAAAAAAAACAAAAGAAGAAGATACAAAAAACGTACTCTCACGTGCCTATCATGTGTCTTCGACTTA
