# Supplementary figures and images for: The structure, organization and radiation of Sadhu non-long terminal repeat retroelements in Arabidopsis species
Source: Mob DNA. 2010 Mar 1;1:10. doi: 10.1186/1759-8753-1-10 (PMC2848041; doi:10.1186/1759-8753-1-10)

A. *Sadhu1*

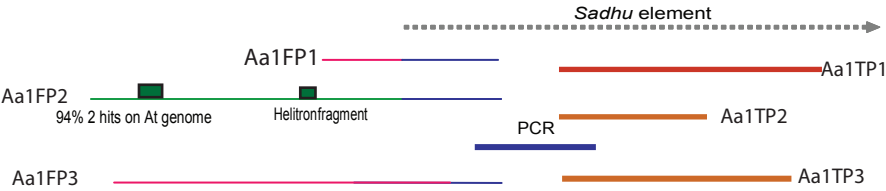

B. *Sadhu3*

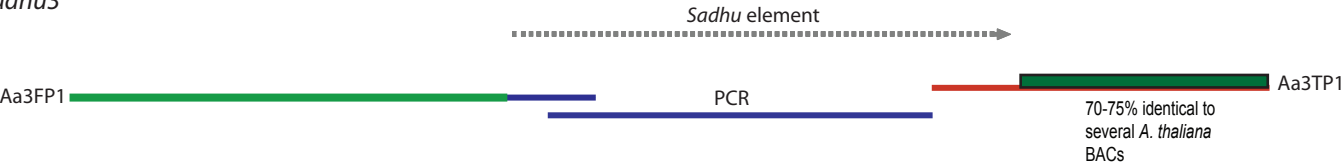

C. *Sadhu5*

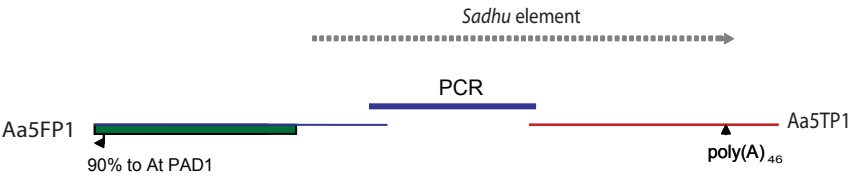

D. *Sadhu8*

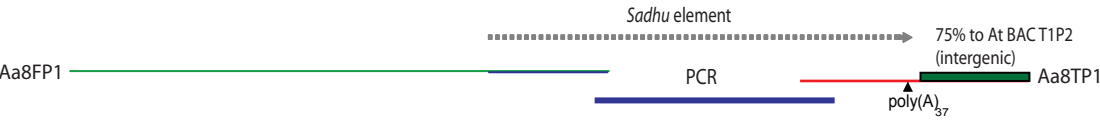

500 bp

Supplement: Additional file 4 — Partial Sadhu elements and flanking genomic sequences identified in Arabidopsis arenosa. Additional file 3 contains diagrams of partial Sadhu elements and flanking genomic sequences identified in A. arenosa. (a) Sadhu1; (b) Sadhu3; (c) Sadhu5; (d) Sadhu8. The scale is indicated. Internal polymerase chain reaction (PCR) sequences used specific primers based on the Arabidopsis thaliana sequence, while 5' and 3' sequences were obtained by thermal asymmetric interlaced (TAIL) PCR (see Table 4 for details). 5' Sadhu sequences are in blue, 3' Sadhu sequences are orange. Gray dotted arrows indicate the extent of Sadhu sequence homology. Features in flanking sequences are marked as green boxes. The inverted arrow in the annotation of the Aa5FP1 clone indicates the direction of transcription of the flanking gene-related sequence. Sadhu5 and Sadhu8 3' sequences feature poly(A) tracts at the Sadhu boundary, consistent with retrotransposition. [file 1759-8753-1-10-S4.PDF]
